# Development of a Theoretical Model for the Price Formation of Agri-Food Products in the Food Supply Chain: A Slovenian Case Study

**DOI:** 10.3390/foods14030415

**Published:** 2025-01-27

**Authors:** Jernej Prišenk, Nejc Zidar, Jernej Turk

**Affiliations:** Faculty of Agriculture and Life Sciences, University of Maribor, Pivola 10, 2311 Hoče, Slovenia; nejc.zidar2@um.si (N.Z.); jernej.turk@um.si (J.T.)

**Keywords:** price formulation, theoretical model, food supply chains, econometric modelling, Slovenia

## Abstract

The main objective of the research was to develop a theoretical model for price construction in the agri-food sector, which should enable better transparency and efficiency of pricing policies in the food supply chain. The central part of the article focuses on the data set, in which the data sources and the economic analysis methodology are presented in detail. Based on this analysis, a theoretical model for pricing was developed, which is presented in more detail in a separate chapter. The model focuses on prices in three sectors (primary production, food processing industry and distribution/retail) of different food and agri-food chains and offers concrete solutions to improve pricing in these chains. The final chapter contains guidelines and recommendations for the practical application of the model with the aim of improving the transparency and effectiveness of pricing policy in the agri-food sector. The overall report represents an important contribution to understanding the behaviour and responsiveness of agri-food systems to specific cost changes and provides useful guidance for policy makers and stakeholders in the agri-food sector.

## 1. Introduction

The food supply chain (also known as the agri-food chain) is a complex system that encompasses all the steps and processes required for the production, processing, distribution and final consumption of food. It is characterised by the fact that it comprises many different players: farmers, companies in the food processing industry, distribution or transport companies, wholesalers, and retailers [1,2] who create added value with their inputs or processing [3,4,5,6,7,8,9,10]. At each stage of the supply chain, products can be modified and sold as new or modified products to other actors in the chain. The prices paid for the products at each stage reflect the value of the inputs, the payment of labour and other factors of production, and the profit margins [11,12,13]. Typically, the various supply chains of agricultural and food products differ according to the number of actors involved or the different organisation of the chains in each country [1,2,8,14,15,16,17]. The prices of the individual inputs in the supply chain can change over time. These changed input prices can be passed on to the next actor in the chain and change the prices of intermediate products or influence the profit margin of the actors involved [14,15,18]. Changes in the production process can affect production costs, prices and/or profit margins [13]. For these reasons, pricing in the food chain is a complex process [19,20,21] in which many factors play a role, from production costs [22,23], seasonality, weather influences, logistics and market conditions to regulation and the strategies of individual companies [7,17,24,25]. Understanding these factors is the key to effective price management and ensuring competitiveness in the market [13,21].

The originality and scientific relevance of this paper is expressed in an organised methodological approach that deals with the structure of the agri-food sectors and provides unique results in terms of the sensitivity of each sector to changes in cost policies related to the production and processing of agri-food products. According to the literature review and previous research, the contribution of the research to existing knowledge lies primarily in the methodological approach, which makes it possible to analyse and identify the types of costs that influence the pricing of food within the individual agricultural and food chains. The innovativeness of the research lies in the fact that it is possible to transfer the results at the micro level (elasticity of price movements of individual types of food in relation to cost changes) to the analysis at the macro level, and thus, to assess the sensitivity of individual sectors within agricultural and food chains to cost changes and the chain as a whole. The additional progress presented in this paper is also evident from a methodological point of view and is related to the improvement of the stationarity test of the data series through the so-called safety test model with a feedback loop.

This article is divided into several main chapters and sub-chapters in which various aspects of pricing are systematically dealt with. The introductory part sets out the basic objectives and the importance of the research, followed by chapters dealing with an overview of best practise in other EU countries, in which different pricing methods are analysed. The central part of the paper deals with the data set, in which the data sources and the economic analysis method are presented in detail. Based on this analysis, a theoretical model for price formation was developed, which is presented in more detail in a separate chapter. The model focuses on prices in three sectors (primary production, food processing industry and distribution/retail) of different food and agri-food chains and offers concrete solutions to improve pricing in these chains.

The final chapter contains guidelines and recommendations for the practical application of the model with the aim of improving the transparency and effectiveness of pricing policy in the agri-food sector. The overall report represents an important contribution to understanding the behaviour and responsiveness of agri-food systems to specific cost changes and provides useful guidance for policy makers and stakeholders in the agri-food sector.

### 1.1. Literature Review

The food price monitoring tool (FPMT) is an instrument for monitoring food prices in the member states of the European Union and is used within the framework of Eurostat. The aim of this tool is to provide accurate and up-to-date data on food prices, allowing market conditions to be analysed, inflation to be monitored and the affordability of food to be assessed. The food price monitoring tool provides information on the evolution of food prices in different parts or at different stages of the food supply chain.

The FPMT is an important tool or criterion for measuring the European economy. Among other things, it helps to determine the inflation rate in different EU countries. Bunte et al. [26] point out that caution is required when comparing price trends between different countries, as there may be differences in product quality or processing methods, for example, or products may be defined differently. It is also difficult to assess price transmission in the chain using indices, as the share of the primary producer’s price in the consumer price can vary from product to product and between countries [27].

In the study by Masten et al. [28], the researchers set themselves the task of developing a tool or methodology for monitoring prices and margins in vertical food supply chains. In their view, the best way to visualise the added value in each chain is to explain the possible drivers of price changes, and most food-price-monitoring tools are flawed because they only monitor the final prices of products by link in the food chain, not the costs. The project used a standardised set of products whose production chains are statistically well validated. The data used in the project were collected at three levels of the food chain (at the level of agricultural production, food processing industry and retail) and related to the prices of products along the food chains and to the production costs of products in individual agricultural and food chains. The entire analysis and research work were carried out on the basis of an adapted methodology, the so-called “food dollar”, which was developed by the American institute USDA-ERS and enables the breakdown of food expenditure into the contributions of the individual segments of the supply chain [29]. The analysis of the Slovenian food in Euros was carried out on the basis of input–output matrices, which are a standard macroeconomic tool for value flows between sectors in the economy and allow us to analyse the structure of the entire economy or only individual sectors. Symmetric I-O matrices link the production (expenditure) side of the economy with the consumption (income) side, allowing researchers to break down the expenditure on a particular product into the contributions of all sectors involved in the production of the analysed product via intermediate input matrices [28]. Based on the modelled breakdown of the Slovenian food in Euros for the year 2009, the authors found that in the overall structure of the final demand for food, the value added percentage of the domestic sectors accounted for 49%, food imports for final consumption for 19%, imports of products for intermediate consumption for 17% and excise duties for 15%. Regarding excise taxes, they emphasised that such a high share of taxes is due to the inclusion of tobacco products in the analysis, which are characterised by a high tax burden. The value added contribution of the domestic economic sectors (direct and indirect contributions) to the total value of food demand was divided between the individual sectors according to their shares: primary agriculture 11%, food processing industry 9%, wholesale and retail trade 15%, transport 2%, real estate and housing 2% and other sectors totalling 10% [28].

While many authors have dealt with the purchasing power and distribution of product pricing in agri-food systems, the essence of this research aims to identify the factors that influence the construction of the price of food, as described in the results of the contribution to the analysis of the level of maximum sensitivity to a change in the price of agri-food products in the case of changes in the level of costs within an individual sector. The econometric analysis was carried out for three different food sectors (dairy products, meat and cereals). Such an approach was not found in the previous literature and the main scientific contribution is to provide a theoretical basis for the food price construction model, which is explained in the discussion and recommendations developed on the basis of the research results.

### 1.2. Hypothesis of the Study

The hypotheses below are exclusively predictions of the authors based on the literature reviewed, experience using the database in Slovenia related to the agri-food sector, and previous, not-very-extensive studies dealing with specific segments related to agribusiness and rural development [30,31,32].

(a) We assume that the economic models developed will successfully identify the dispersion of the different types of costs that affect the pricing of food.
(b) We assume that the results of the elasticity of food price development in relation to cost changes in the food chains will follow meaningful and current trends in cost development in agricultural production and the processing industry.
(c) We assume that with the results obtained at the micro and macro levels, we will be able to contribute to the innovation to understand the sensitivity of each sector in the food chains and that these results will be useful for the timely development of new support measures in the agricultural sector.

## 2. Materials and Methods

### 2.1. Selection of the Appropriate Type of the Model

Various forms of standard econometric models (linear, logarithmic and combinations of both) were tested as part of this study. The decision as to which form of model is most suitable was made on the basis of the VAR model (vector autoregressive models) and the standard forms of econometric models. VAR models are best suited for multivariate time series in which dynamic interactions play an important role. Standard econometric models (linear, logarithmic and combinations of both) are better suited for hypothesis testing and causal analyses of cross-sectional or time series data. Since clear research hypotheses were established, the focus of the research was on calculating the elasticity and relationship of interactions between individual variables and other characteristics shown in Table 1, which we chose to use as standard econometric models.

The construction of the theoretical model for the construction of the price is presented in three parts, which follow the course of the research work on the project itself.

**Figure 1:**
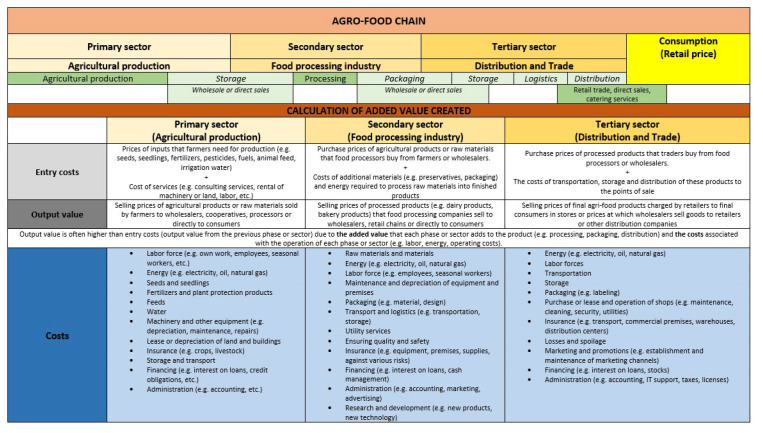
Schematic representation of the food or agri-food chain with definitions of input and total costs and output value.

In the first part, the food chain or agri-food chain is presented schematically, which is logically divided into three sectors (primary, secondary, tertiary) (Figure 1). Within each sector, the most typical activities that are usually carried out at each stage of the chain are listed [35]. These activities add value to individual agri-food products, which is the fundamental purpose of the entire agri-food chain [5,35]. To better understand this concept of added value, it is crucial to know the contribution of each sector, which is indirectly expressed through the input costs and the output value of each product as it passes from one sector to another [1,5,36,37]. Input costs are characterised by the fact that they usually represent the production value of the previous sector or stage in the chain itself. The production value usually expresses the added value that the individual sector adds to the food product, as well as the total costs associated with the operation of the individual sector [9,10,35]. This is also the main reason why the production value is usually higher than the input costs. The schematised representation also includes a number of costs that are typical for an individual sector or stage of the agri-food chain. These have a significant influence on the production value of an individual agri-food product in an individual sector, i.e., by transferring the price via the input costs and the production value in the chain, they are also reflected in the final price (retail price) of an individual agri-food product.

**Figure 2:**
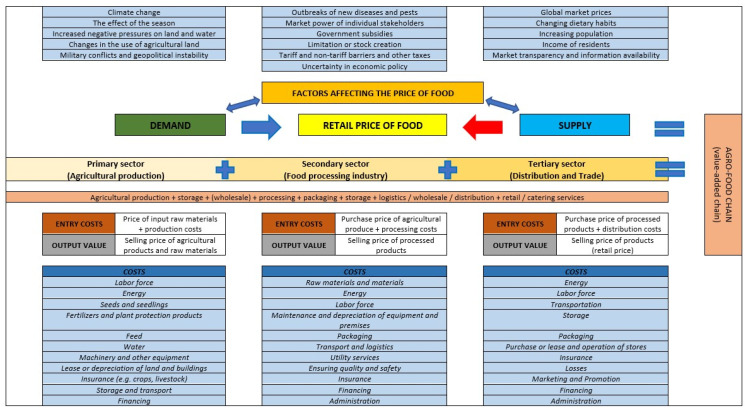
Schematic representation of the food or agri-food chain with defined costs and identified factors that influence the retail price of a food or agri-food product.

In the second part, a schematic illustration shows the influence of supply and demand on the retail price of food, with the price generally falling when supply increases and demand remains unchanged (red arrow), and the retail price generally rising when demand increases and supply remains unchanged (blue arrow) (Figure 2) [38,39,40,41,42,43,44]. The supply of food and individual agricultural and nutritional products depends primarily on the functioning of the individual agricultural and nutritional chains, which can be divided into three basic sectors for simplicity’s sake. The primary sector is agricultural production, the secondary sector is the food industry or processing, and the tertiary sector is distribution and trade. The retail price of a single agricultural and food product corresponds to one-sixth of all activities organised in the chain that add value to the same product across different sectors. The value added in the individual areas of influence can best be visualised with the help of input costs and output values. However, since the retail price of an individual agri-food product depends not only on the functioning of the individual agri-food chain, but also on the conditions of the national and international market (supply and demand), the identified factors that influence supply and demand and that directly or indirectly influence the retail price of an individual agri-food product are also included [41,42,43,45,46,47,48,49,50]. The identified factors influencing the price of an agri-food product and the defined (total) costs in each sector of the agri-food chain as well as the concept of input costs and production value were key to the creation of a theoretical (econometric) model to calculate the individual price of an agri-food product or a group of agri-food products [14,41,42,44,47,48,49,50,51,52,53,54,55,56].

**Figure 3:**
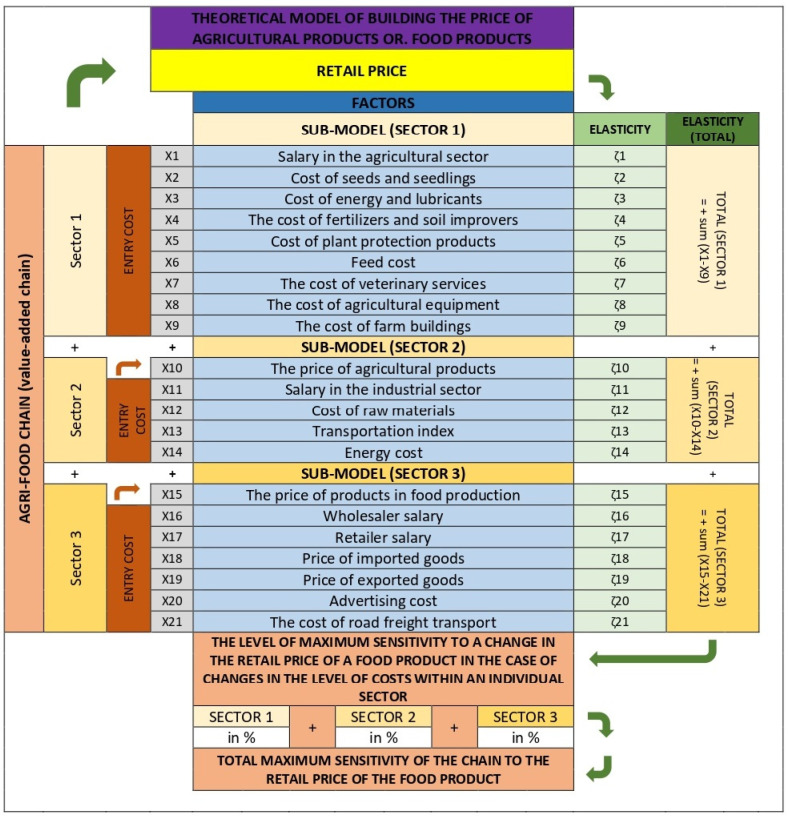
Schematic representation of the theoretical (econometric) model for the formation of the price of food or agricultural and nutritional products.

In the third part, the theoretical (econometric) model for the formation of the price of agricultural and food products is presented schematically (Figure 3). In the first part, the model consists of three sectors or three separate sub-models, similar to a simplified food chain, which are linked or combined in the second part. Each of the three sub-models represents one of the three sectors in the respective agri-food chain (primary, secondary, tertiary), which enables the definition of the importance or influence of each sector in the chain on the retail price of an individual agri-food product or a group of agri-food products. Each sub-model defines the factors that influence the business in each sector or in the key activities of each sector. In defining the factors to be included, care has been taken to maximise the number of factors that represent or influence costs in each sector. An essential condition for the inclusion of a single factor was the availability of an appropriate and sufficiently long series of statistical data describing the factor itself (numerically). As this condition was not met, some theoretically important factors were also excluded from the model itself. For some factors, data were included in the form of actual or absolute values (e.g., the level of gross wages in a single month), whereas for others, data were included in the form of indices indicating the monthly change (value in the reporting month compared to the previous month). An econometric analysis was then carried out for each sector or sub-model in which the impact of each factor on the retail price of the agricultural and food product was analysed. This influence is expressed by the calculated elasticities for the individual variables (factors) included. All three sub-models are concerned with analysing the influence of factors that are important in each sector on the retail price of food. Such a model makes it possible to define the degree of maximum sensitivity to changes in the retail price of an agri-food product or a group of agri-food products in the event of changes in the cost level within a single sector. Just as the retail price reflects the sum of all activities in the individual sectors of the agri-food chain, in the theoretical model, the influence of the individual factors is first expressed within the individual sector in the form of weights (converted from calculated elasticities), and then also as the sum of the weights of all three individual sectors in the final theoretical model for the formation of the price of agri-food products.

### 2.2. Specification of the PRICE/PRICE Econometric Sub-Model—Basic Model

Following the above, we wanted to use the results of the developed econometric sub-models to show the influence of individual identified factors on the price of agricultural and food products or broader basic groups of agricultural and food products. To better understand the influence of each broader sector within each agri-food chain, we categorised the factors into three groups, namely the factors that have a decisive influence on the primary sector (the agricultural sector), the secondary sector (the processing sector or food industry), and the tertiary sector (the distribution sector or shops). For each individual sector, we developed an econometric sub-model that was linked to the change in the (retail) price of the agricultural and food product as a function of the change (quantities, prices) in the factors included. In total, 12 functional forms of econometric models were estimated for each agri-food product or group of agri-food products (i.e., a basic sub-model for each sector of the agri-food chain × 3 sectors in each agri-food chain × 4 different models of functional forms (LIN, LOG, LIN-LOG and LOG-LIN)).

The time series included differed according to the availability of data, the collection methods for the individual food products within the sectors, the costs and the appropriateness of use and inclusion in the individual models.

The variables or input data included in the sub-models for the primary sector (primary production or agricultural sector) are as follows:

*Y*—Price of food (price of consumer goods, in indices).

*Ya*—Total food; *Yb*—bread and cereal products; *Yc*—meat; *Yd*—milk, cheese and eggs.

*X1*—Average gross wage in each agricultural activity (actual amount in Euros).

*X1a*—Agricultural production and hunting and related services; *X1b*—cultivation of cereals [except rice], pulses and oilseeds; *X1c*—animal husbandry; *X1d*—dairy processing.

*X2*—Costs of seeds and seedlings (intermediate consumption in agriculture, in indices).

*X3*—Costs of energy and lubricants (intermediate consumption in agriculture, in indices).

*X4*—Costs of fertilisers and soil improvers (intermediate consumption in agriculture, in indices).

*X5*—Costs of plant protection products (intermediate consumption in agriculture, in indices).

*X6*—Feed costs (intermediate consumption in agriculture, in indices).

*X7*—Costs of veterinary services (intermediate consumption in agriculture, in indices).

*X8*—Costs of agricultural equipment (intermediate consumption in agriculture, in indices).

*X9*—Costs of agricultural buildings (intermediate consumption in agriculture, in indices).

We have collected a time series of 216 monthly data spanning eighteen years between January 2005 and December 2022.

The variables or input data included in the sub-models for the secondary sector (processing sector or food industry) are as follows:

*Y*—Price of food (price of consumer goods, in indices).

*Ya*—Total food; *Yb*—bread and cereal products; *Yc*—meat; *Yd*—milk, cheese and eggs.

*X1*—Prices of agricultural products from producers (in indices).

*X1a*—Agriculture—combined, with fruit and vegetables; *X1b*—cereals; *X1c*—animals for slaughter; *X1d*—milk, cow.

*X2*—Average gross wage in the individual activity of the economic sector (actual amount in Euros).

*X2a*—Manufacture of food products; *X2b*—manufacture of bakery and pasta products; *X2c*—manufacture of meat and meat products; *X2d*—milk processing.

*X3*—Raw material costs (price of industrial products for manufacturers on the domestic market, in indices).

*X4*—Transport index (defined in point 4.3.6).

*X5*—Cost of electricity, gas and other fuels (prices of consumer goods, in indices).

We have collected a time series of 156 monthly data covering thirteen years between January 2010 and December 2022.

The variables or input data included in the sub-models for the tertiary sector (distribution or trade sector) are as follows:

*Y*—Price of food (price of consumer goods, in indices).

*Ya*—Total food; *Yb*—bread and cereal products; *Yc*—meat; *Yd*—milk, cheese and eggs.

*X1*—Average gross wage in wholesale trade in each chain (actual amount in Euros).

*X1a*—Wholesale of food, beverages and tobacco; *X1b*—wholesale of cereals, tobacco, seeds and animal feed; *X1c*—wholesale of meat and meat products; *X1d*—wholesale of milk, milk products, eggs, edible oils and fats.

*X2*—Average gross wages and salaries in retail trade in each chain (actual amount in Euros).

*X2a*—Retail sale in specialised shops of food, beverages and tobacco; *X2b*—retail sale in specialised shops of bread, bakery products, pasta and confectionery; *X2c*—retail sale in specialised shops of meat and meat products.

*X3*—Average prices of imported goods (in indices).

*X3a*—Food and live animals; *X3b*—cereals and cereal products; *X3c*—meat and meat products; *X3d*—dairy products and eggs.

*X4*—Average price of exported goods (in indices).

*X4a*—Food and live animals; *X4b*—cereals and cereal products; *X4c*—meat and meat products; *X4d*—dairy products and eggs.

*X5*—Prices of industrial products at manufacturers in food production (in indices).

*X6*—Cost of services for manufacturers in advertising (in indices).

*X7*—Cost of services for manufacturers in road haulage and removal activities (in indices).

We collected a time series of 216 monthly data spanning eighteen years between January 2005 and December 2022. Quarterly data between 2006 and 2022 were used for the producer price of services’ data.

The main purpose of these econometric sub-models was to determine how, or how intensively, individual theoretically defined factors relating to prices and costs in each sector of the agri-food chains influence the change in the final selling price of agri-food products in four basic food categories or groups of agri-food products [57].

### 2.3. Specification of the Econometric Model CONSUMPTION/PRICE—Test Model

The purpose of the second step of the econometric analysis was to additionally check the results and the correctness of the basic econometric sub-models (price/price sub-models). Replacing the dependent variable in the basic econometric models while retaining all independent variables proved to be one of the most suitable theoretical options. To determine the appropriateness or correctness of the calculated elasticities and the response of the dependent variable to changes in individual independent variables, we had to replace the dependent variable in such a way that, at least theoretically, elasticities with opposite (negative) values are calculated with the same independent variables. As an example, we took the theoretical reaction of supply and demand to price changes [58,59,60].

When prices change, it is normally expected that the demand for an item will fall when the price rises, or that the demand for the item will rise when the price falls, because the relationship between price and quantity demanded is negative (law of demand). The reverse is true for supply: if the price rises, the quantity offered also rises, which results from the positive relationship between price and quantity offered (law of supply) [61].

In our case, this means that the value of the price of an agricultural and food product or a group of agricultural and food products (dependent variable) should increase if the values of the independent variables increase (the calculated elasticities for the individual independent variables should be positive). However, if the value of the price of an agri-food product or group of agri-food products (dependent variable) is replaced by the level of consumption per agri-food product or group of agri-food products, the opposite response is expected, i.e., an increase in the values of the independent variables (e.g., prices, wage levels, etc.) decreases the consumption per agri-food product or group of agri-food products (the calculated elasticities for the individual independent variables should be negative). It was concluded that the dependent variable (the price of an agricultural product or group of agricultural products) should be replaced by consumption (monthly consumption of an agricultural product or group of agricultural products).

Due to gaps in the statistical data, where we did not collect data on the monthly consumption of food, cereals and cereal products, meat and milk, cheese and eggs, we had to define monthly consumption based on the available statistical data. We obtained data on the total annual domestic consumption of cereals, meat and milk. These data were adjusted to the monthly level using a defined weighting that takes into account the main factors influencing consumption and a normalisation procedure. We identified the following key factors that are adequately supported by statistical data and influence consumption: the price of individual food items, the inflation rate in the country, the average monthly salary in the country and the proportion of the working population in the country.

The weighting comprised the sum (expressed as a percentage) of the monthly changes in the four factors included that influence food consumption. We included monthly data (month/month indices) on the following topics:

- Consumer price index for cereals and cereal products; meat; milk, cheese and eggs;
- Inflation index;
- Average monthly gross wages in Slovenia;
- Employed population in Slovenia.

Normalisation is the process [62] of adjusting the values of different data points in a data set to a common scale without changing the differences in the range of values between the data points. The purpose of normalisation is to make data comparable, especially if they come from different sources or are measured in different units.

In our case, the normalisation process for the individual monthly consumption data consisted of 6 steps:

1. The annual domestic consumption data for cereals, meat and milk were used as the basis for all 12 months (same data for all 12 months);
2. The weighting value for each individual month was calculated by adding the monthly index changes (in %) of all 4 included factors;
3. We multiplied the two data from points 1 and 2 and subtracted the product from the data from point 1;
4. The final difference from point 3 was divided by the product of point 3;
5. We added the resulting quotient with the value −1 and obtained the difference, which represents the monthly change in consumption;
6. We multiplied the difference from point 5 by the data from point 1 and obtained the monthly consumption data.

The monthly consumption data calculated using the normalisation procedure were either included as a new dependent variable (new *Y* in previously created econometric models, or we replaced the old dependent variable (*Y*—food price) with a new one (*Y2*—food consumption).

### 2.4. Summary of the Theoretical Explanation of the Combination of Two Models (Basic and Test Models)

It is expected that the calculated elasticities in the PRICE/PRICE and CONSUMPTION/PRICE models are exactly opposite [58,59]. In the PRICE/PRICE model, an increase in the price of one of the production inputs is expected to be reflected in an increase in the (final) retail price of the product (+/+); or, on the contrary, if the price of one of the production inputs decreases, the (final) retail price will also increase, while the price of the product will decrease (−/−) [37]. In the case of the CONSUMPTION/PRICE model, it is expected that if the price of a certain production input increases, the demand for a certain product will decrease (+/−); or, on the contrary, if the price of the production input decreases, the demand for a certain product will increase (−/+). An increase in the price of production inputs can discourage producers from producing, which affects the volume or quantity of supply, which usually decreases in such a situation. Lower supply with unchanged demand usually leads to an increase in market prices. A reduction in the price of inputs can be an important signal for producers to increase their production, which also increases supply on the market. Increased supply with unchanged demand usually leads to a fall in product prices on the market.

### 2.5. Testing Model Approaches

This chapter explains the procedure for testing the specified models under study with regard to the most common statistical phenomena or so-called “diseases” that accompany any econometric model developed. It is important that the problems that affect the accuracy of the predictive power of the model and the appropriateness of the model specification are manageable and within normal limits. The testing of the various statistical phenomena in the models developed in this research are indicated in the following points. The summaries of the tests are presented in Table 2, Table 3 and Table 4, while the other statistical tests are described in the text in the results of the interpretation of the individual models within the agricultural and food chain sectors.

(A) Stationarity Test: As already described in Section 2.1, the choice was made between the VAR model and the test of standard forms of models. The VAR model assumes stationarity of the time series (or the use of differences if they are not stationary), while standard econometric models do not always require stationarity; this depends on the model (e.g., linear regression does not require strong stationarity). In statistics, an augmented Dickey–Fuller (ADF) test examines the null hypothesis to determine whether a unit root is present in a time series sample. The ADF test was performed to confirm the stationarity of the data. The data collected were used in all sectors; the cereal sector is shown in Table 2.

It should be emphasised that time series data are stationary in the vast majority of cases. The series are stationary and can be used for analysis without further processing. In the rare cases where the value of the ADF test is above the critical value, the prediction of variables with the results of the test models was excluded, or elasticity values of zero or close to zero were calculated for them. The test models served as an additional “safeguard model” (consumption/price model) to avoid incorrect predictions (presented in section results and discussion), which is not possible with only stationary test results.

(B) Inadequate Residual Analysis: Autocorrelation in the residuals violates the assumption of independence and leads to biassed standard errors and invalid hypothesis tests. We used the Durbin–Watson test to test the model for autocorrelation. The Durbin–Watson (DW) test is a statistical test used in regression analysis to determine the presence of autocorrelation (especially first-order serial correlation) in the residuals of a regression model. Autocorrelation is present when the residuals (errors) are not independent but are correlated with each other. DW ≈ 2 indicates no autocorrelation; d < 2 indicates positive autocorrelation, and d > 2 indicates negative autocorrelation. Table 3 contains the results of the DW tests. It is important that there is no autocorrelation in the model or that it is limited to a reasonably low level. The values in Table 3 show the corresponding estimates for the normal state of autocorrelation in the models. The value of 2 can only be talked about in an “ideal world” and is impossible to expect when modelling socio-economic situations in the market (as in the complexity of this research). Based on the DW test values, we can say that the models developed are very good and have exceeded our expectations in certain segments (such as the LIN model in the tertiary meat sector and the LIN-LOG model in the primary and secondary dairy sector).

(C) R^2^ Value and Explanatory Power: We emphasise that a low R^2^ value does not necessarily invalidate the model, especially if it performs well in prediction tasks or matches theoretical expectations. A low R^2^ indicates that the model does not explain much of the variance, but it does not necessarily invalidate the study. Some data inherently exhibit a high degree of noise or randomness, especially for highly volatile time series such as prices. Since the models showed no autocorrelation and the multicollinearity tests showed no serious problems, we assume that the predictive power of the models is reasonable as they follow certain established hypotheses, understandable arguments of the results of agricultural economic laws and the discussion of natural phenomena in the agri-food sector.

A study [63] examining the impact of relationship quality on supplier performance in food supply chains reported low R^2^ values. This outcome suggests that factors beyond relationship quality significantly affect supplier performance. The authors note that elements such as supplier capabilities, market conditions, and external economic factors likely play substantial roles, which were not fully captured in the model.

**Figure A1:**
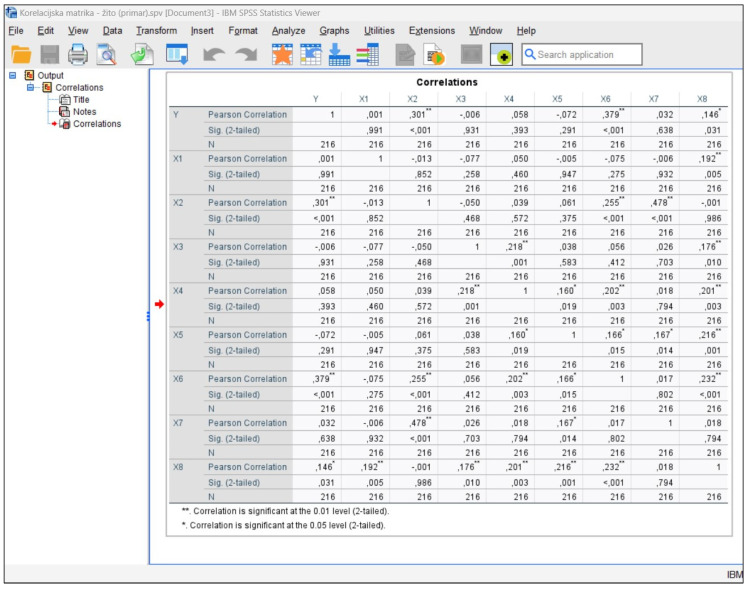
Results of correlation matrix—cereal primary sector.

**Figure A2:**
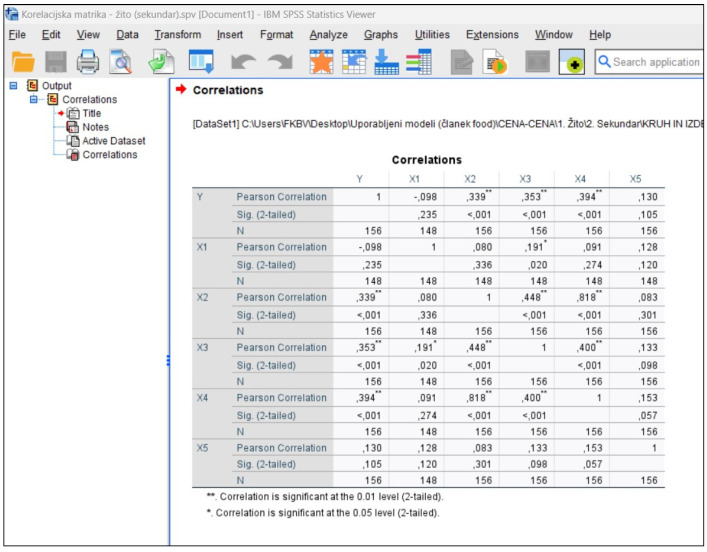
Results of correlation matrix—cereal secondary sector.

**Figure A3:**
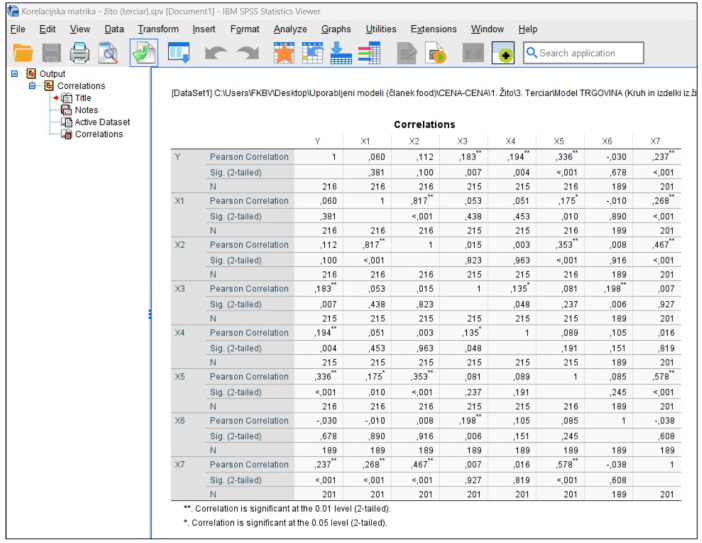
Results of correlation matrix—cereal tertiary sector.

**Figure A4:**
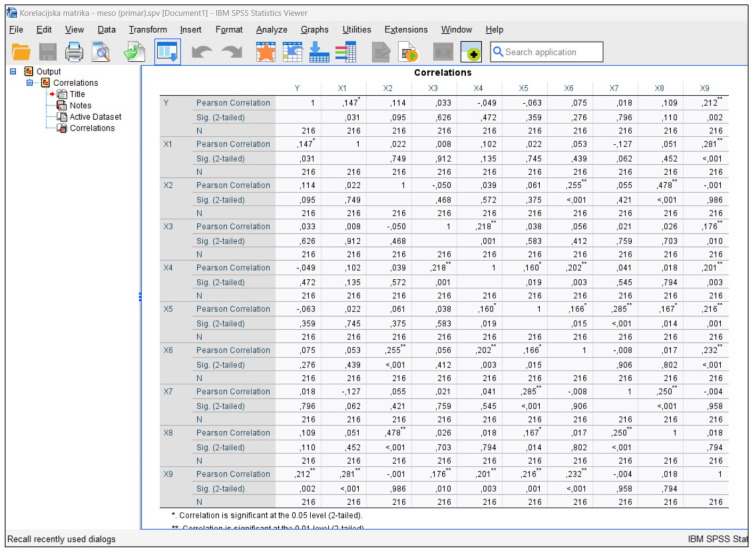
Results of correlation matrix—meat primary sector.

**Figure A5:**
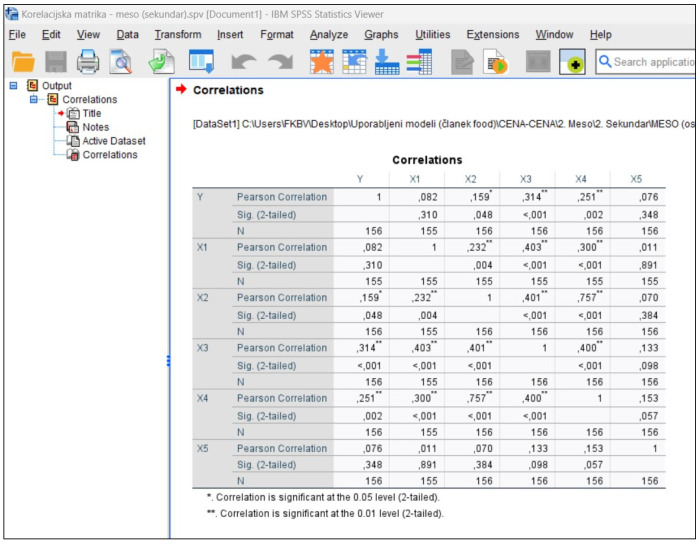
Results of correlation matrix—meat secondary sector.

**Figure A6:**
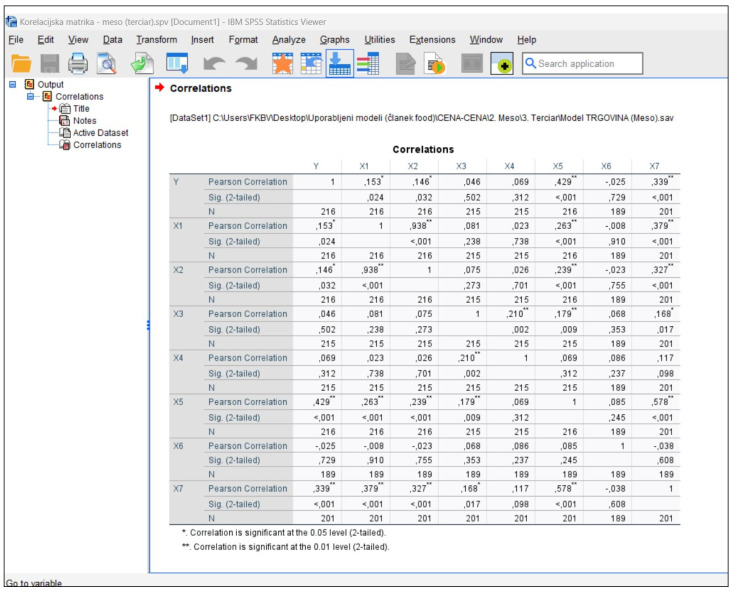
Results of correlation matrix—meat tertiary sector.

**Figure A7:**
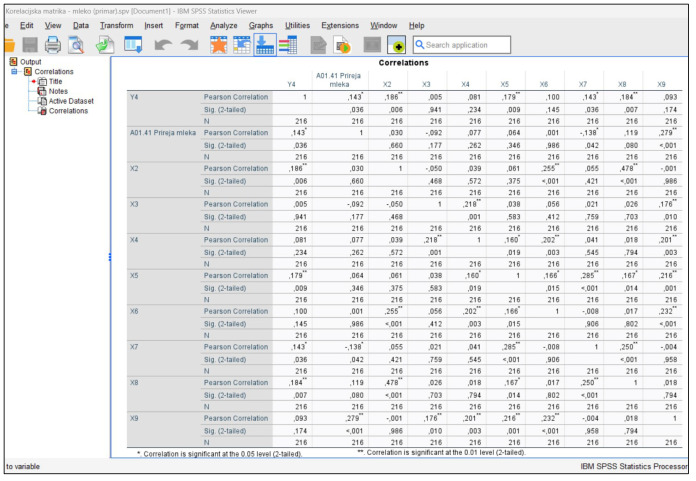
Results of correlation matrix—dairy primary sector.

**Figure A8:**
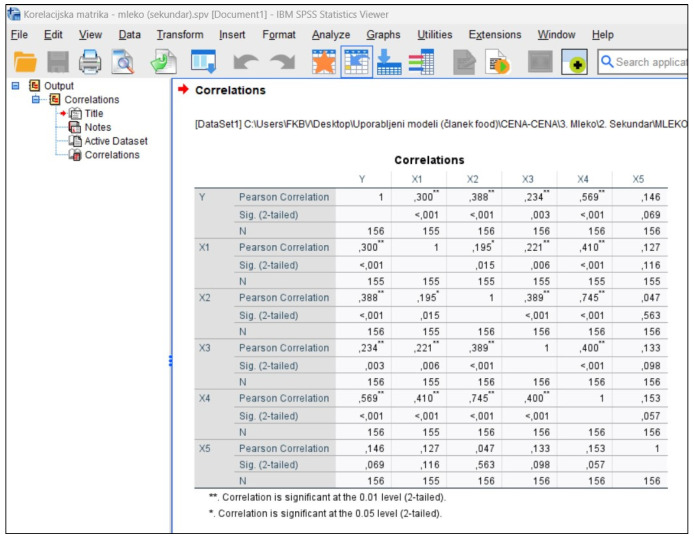
Results of correlation matrix—dairy secondary sector.

**Figure A9:**
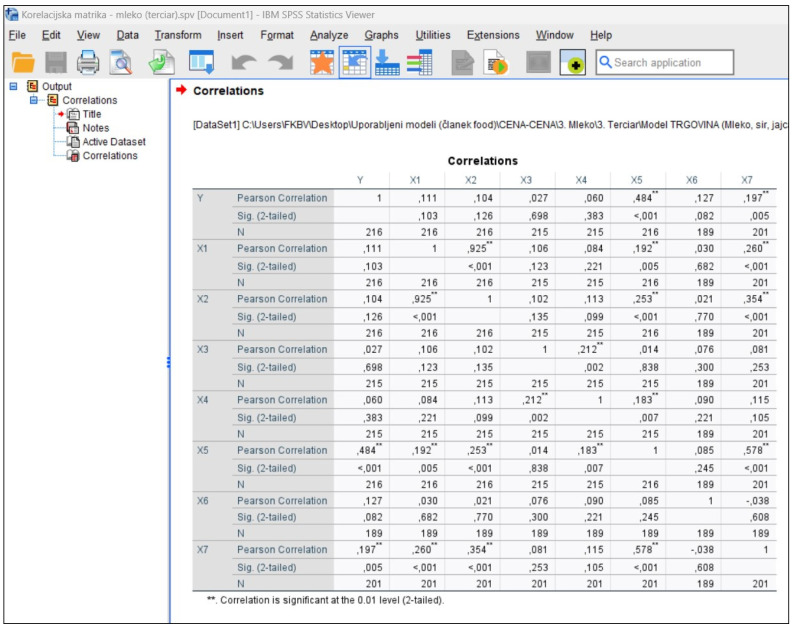
Results of correlation matrix—dairy tertiary sector.

The author of ref. [64] discusses how, in applied microeconomics, including agricultural economics, R^2^ values ranging from 0.05 to 0.30 are common. This reflects the substantial unobserved heterogeneity present in such data. The focus in these studies is often on identifying causal relationships rather than solely on the predictive power of the model.

(D) Variable Selection and Multicollinearity Issues: The concern about variable selection and multicollinearity highlights a critical omission in the study. Including a large number of input variables without considering possible multicollinearity can significantly affect the stability, interpretability and reliability of the regression results. The best way to diagnose multicollinearity is to use the correlation matrix (examine the pairwise correlations between the predictors; values > 0.7 or <−0.7 indicate potential problems). Figure A1, Figure A2, Figure A3, Figure A4, Figure A5, Figure A6, Figure A7, Figure A8 and Figure A9 show the correlation matrix. The Pearson correlation is therefore suitable for a quick assessment, as two variables are in a close linear relationship with each other, and for testing whether this relationship is significant.

The results show that there are no critical relationships between the variables at a statistically significant value of 0.01, except in the case of the tertiary sector, which shows a possible relationship between wages in the sectors and wages at the national level, which does not prove a possible multicollinearity in the model.

In addition to the correlation matrices, we show the VIF values in Table 4. The variance inflation factor (VIF) is a measure for assessing multicollinearity in the regression analysis. It indicates the extent to which the variance of the regression coefficient is inflated by multicollinearity between the independent variables.

Interpretation of the VIF value:

- VIF = 1: No multicollinearity (the variable is not correlated with other independent variables).
- 1 < VIF ≤ 5: Moderate multicollinearity, generally acceptable.
- VIF > 5: High multicollinearity, may need to be investigated.
- VIF > 10: Very high multicollinearity, problematic and the variable may need to be removed or treated (e.g., by regularisation or dimensionality reduction).

## 3. Results

### 3.1. Theoretical Model of Price Formation—Cereal Sector

The model consists of three sectoral sub-models (primary, secondary, tertiary) with which we have analysed the influence of the factors included, which are characteristic of each individual sector of the cereal agri-food chain, on the retail price of bread and cereal products. In the sub-model for the primary sector (agricultural production), we have included factors related to labour costs in grain production (gross wages in the agricultural activity of grain, legume and oilseed production) and changes in the costs of agricultural inputs (seeds and seedlings, energy and lubricants, fertilisers and soil improvers, etc.). With these data, we have captured most of the costs that are typical of the primary sector of the food chain or that affect the level of the output price in this sector. The sub-model for the secondary sector includes data on the evolution of the price of cereals among agricultural producers (which represents the starting price of the primary sector), data on labour costs in the production of bakery and pasta products and data on the evolution of the costs of raw materials, energy and transport (in indices). Based on the analysis of the functioning of the secondary sector, especially in terms of value added and typical costs, we have captured most of the important influences on the exit price at this stage of the chain with the factors included. The sub-model for the tertiary sector, which includes distribution and trading activities, contains data on the change in the price of industrial products in food production (the data represent the exit price of the secondary sector, which is not entirely accurate at this point, as it is the change in the price of food industry products as a whole, not just grain products), data on wholesale and retail labour costs for grain and grain products, data on the change in the average import and export price of grain and grain products, and data on the change in service costs (advertising and road freight transport). In all three sectors, we wanted to assess the impact of these included factors on the retail price of bread and cereal products (consumer price index) or to determine the response of the retail price of bread and cereal products to changes in the included factors within each sector.

#### 3.1.1. Econometric Sub-Models for the Primary Sector (Combination of the PRICE/PRICE and CONSUMPTION/PRICE Models)

Here, we present the properties of the econometric models we used to analyse the dependence of the change in the retail price of bread and cereal products on the change in wages in the production of cereals, pulses and oilseeds and on the change in the costs of agricultural inputs. The functional forms of the econometric models take the form of the equations in Table 5.

All four functional forms of the model are very similar in terms of the statistical and econometric test results. The values of the coefficient of determination (R^2^) are similar for all functional forms of the model, which means that the various transformations have not significantly improved the explanatory power of the model. R^2^ is 21.3%, which means that the model explains about 21% of the variability of the dependent variable. The average value of the Durbin–Watson test for all functional forms of the model is around 2.349 and therefore close to 2, which indicates that there is no significant negative autocorrelation in the model. The standard error varies depending on the functional form of the model and indicates how far away the individual data are from the regression line on average. The F-statistic is statistically significant (*p* < 0.001), which confirms that the regression model is statistically significant and that there is a relationship between the independent and dependent variables. Three variables (*X2*, *X5*, *X6*) are statistically significant (their *p*-value is less than 0.05) or that they significantly influence the dependent variable *Y*. In addition, however, the variables *X7* and *X8* (whose *p*-value is greater than 0.05) are conditionally statistically significant.

Elasticity calculations show that a 1% increase in the average wage in grain production and changes in the costs of energy products and means of soil improvement do not significantly affect the change in prices for bread and grain products. Changes in the cost of seeds and seedlings have a greater positive impact on the change in the price of bread and cereal products (if the cost of seeds and seedlings increases by 1%, the price of bread and cereal products increases by 0.20%).

As shown in the specification of the econometric model CONSUMPTION/PRICE, we replaced the dependent variable in all three models presented below, while all independent variables remained unchanged. The dependent variable, which in the basic models was represented by data on the change in the price of bread and cereal products, was replaced by data on the consumption of bread and cereal products, which we defined ourselves using the available data.

As the most appropriate functional form of the model for the primary sector (agricultural production sector), we have chosen a logarithmic model (LOG model) with a coefficient of determination (R^2^) of 0.053, which means that the model explains only 5.3% of the variability of the dependent variable. There is no autocorrelation in the model as the value of the Durbin–Watson test is close to 2, exactly 1.97. The value of the F-statistic is statistically insignificant or insignificant (greater than 0.05), which indicates that the regression model is statistically insignificant and that there is a relatively weak influence of the individual independent variables on the dependent variable. The calculated elasticities for the individual independent variables show that positive (negative in nature) changes in the costs (price increases or costs) for fertilisers and soil improvement agents, animal feed and agricultural buildings influence the decline in the consumption of bread and cereal products. From the elasticities calculated, it can be concluded that the increase in prices or costs in agricultural production influences the increase in supply for bread and cereal products.

By replacing the dependent variable in the CONSUMPTION/PRICE models, we have reached a situation in which, at least theoretically, the values of the calculated elasticities for the individual variables should be opposite (+/−) to those in the base model (Table 6). At this point, the value of the calculated elasticity for the change in the cost of seeds and seedlings in the PRICE/PRICE model was positive, which is theoretically to be expected and means that if the cost of seeds and seedlings increases, the retail price of bread and cereal products would also increase. However, due to the positive value of the calculated elasticity in the CONSUMPTION/PRICE model, which contradicts expectations and the established rule according to which the calculated elasticities of the opposite values (+/−) should be calculated, this variable was excluded from the joint model for price construction.

#### 3.1.2. Econometric Sub-Models for the Secondary Sector (Combination of the PRICE/PRICE and CONSUMPTION/PRICE Models)

Here, we present the properties of the econometric models we used to analyse the dependence of the change in the retail price of bread and cereal products on the following factors: changes in grain prices among producers; wage changes in the production of bakery and pasta products; changes in the costs of raw materials; changes in the costs of energy products; and changes in the value of the transport index. The functional forms of the econometric models take the form of the equations in Table 7.

All four functional forms of the model are very similar in terms of the statistical and econometric test results. The coefficient of determination (R^2^) values for all functional forms of the model are around 0.145, which is relatively low and indicates that the model can explain around 14.5% of the variability of the dependent variable. The value of the Durbin–Watson test is around 2.463 in all functional forms of the model and deviates slightly from the value 2, which indicates that the model has a slight negative autocorrelation. The standard error varies depending on the functional form of the model and indicates how far the individual data are on average from the regression line. The F-statistic is statistically very significant or significant (less than 0.001), which indicates that the regression model is statistically significant and that there is a relationship between the independent and dependent variables. Three variables (*X1*, *X3*, *X4*) are either statistically significant (their *p*-value is less than 0.05) or they significantly influence the dependent variable *Y*.

The calculated elasticities are very similar in all four functional forms of the model. The calculated elasticities show that an increase in the average wage in the production of bakery and pasta products and a 1% change in energy costs have no influence on the change in the price of bread and cereal products. This is true for the price/price model, while the test model explains the agronomic regularity and the appropriateness of the response to market conditions in the energy sector, as it predicts that an increase in the price of energy products would be reflected in a 5% decrease in the demand for bread and bakery products. We can therefore assume that this is an indirect increase in the price of bread and bakery products (Table 7). A change in the price of grain for producers has only a minimal impact on the change in the price of bread and cereal products (if the price of grain for producers increases by 1%, the price of bread and cereal products increases by 0.01%) and a change in the value of the transport index (if the value of the transport index increases by 1%, the price of bread and cereal products decreases by 0.01%). A change in the cost of raw materials has a greater impact on the change in the price of bread and cereal products (if the cost of raw materials increases by 1%, the price of bread and cereal products increases by 0.26%). In the case of the influence of all independent variables on the dependent variable, this is an inelastic change in the dependent variable (prices for bread and cereal products), as the calculated values do not exceed 1%.

In the models for the secondary sector (food industry), the linear-logarithmic form (LIN-LOG model) proved to be the most suitable functional form of the model, with a coefficient of determination (R^2^) of 0.070, which means that the model explains 7% of the changes in the dependent variable. A Durbin–Watson test value close to 2 (2.08) indicates that the model has no autocorrelation, which is an important sign for the use of the model itself. The value of the F-statistic is 0.063, which is slightly above the upper limit (0.05), indicating that the model is not statistically significant. The calculated elasticities for the individual independent variables show that positive changes in costs (price or costs increases) for transport and energy products and in labour costs in the manufacturing industry influence the decline in the consumption of bread and cereal products. From the elasticities calculated for all other variables included, it can be concluded that the increase in prices or costs in the processing sector of the cereal agri-food chain influences the increase in demand for bread and cereal products.

Similarly to the sub-model for the primary sector, the value of the calculated elasticity for the change in commodity costs in the PRICE/PRICE model was positive, which is theoretically to be expected and means that the retail price of bread and cereal products would also rise with an increase in commodity costs. However, similarly to the sub-model for the primary sector, due to the positive value of the calculated elasticity in the CONSUMPTION/PRICE model, this variable was excluded from the joint model for price construction, as the results of the calculated elasticities did not match the theoretical assumptions, according to which they should have calculated elasticities with opposite values (+/−) (Table 8).

#### 3.1.3. Econometric Sub-Models for the Tertiary Sector (Combination of the PRICE/PRICE and CONSUMPTION/PRICE Models)

Here, we present the properties of the econometric models used to analyse the dependence of the change in the retail price of bread and grain products on the following factors: changes in the level of wages in wholesale trade of grain, tobacco, seeds and animal feed; changes in the level of wages in retail trade in specialised shops of bread, biscuits, pasta, sugar products; changes in the average import and export price of grain and grain products; changes in the prices of industrial products in food production; and changes in the costs of services in advertising and the costs of services in road freight transport and moving activities. The functional forms of the econometric models take the form of the equations in Table 9.

All four functional forms of the model are very similar in terms of the statistical and econometric test results. The coefficient of determination (R^2^) values are approximately 0.173, which means that the model explains 17.3% of the variance of the dependent variable *Y*. The value of the Durbin–Watson test is approx. 2.378 for all functional forms of the model and deviates slightly from the value 2, which indicates that there is a slight negative autocorrelation in the model. The standard error varies depending on the functional form of the model. The F-statistic is statistically significant or significant (less than 0.05) in all functional forms of the model, which indicates that the regression model is statistically significant and that there is a relationship between the independent and dependent variables. Variables *X4* and *X5* (in the LIN and LOG-LIN functional forms) are statistically significant, as their *p*-value is less than 0.05. Variables *X3* and *X6* are conditionally statistically significant, and their *p*-value is greater than 0.05.

Elasticity calculations show that an increase in the average wage in wholesale (wholesale of cereals, tobacco, seeds and animal feed) and retail (retail of specialised shops for bread, bakery products, pasta, sugar products) has no effect on the changes in the prices of bread and cereal products. A minimal or negligible impact can be observed in the changes in the average price of imported and exported grain and grain products. An increase in import and export prices for grain and grain products of 1% each increases the price of bread and grain products by 0.01%. A greater influence on the change in the price of bread and cereal products is exerted by the change in the prices of industrial products at food manufacturers (if the prices of industrial products at food manufacturers increase by 1%, the price of bread and cereal products increases by 0.51%), the cost of producer services in advertising (if the cost of producer services in advertising increases by 1%, the price of bread and cereal products decreases by 0.07%, which is somewhat contrary to the expectations of the research) and the costs of producer services in road transport and moving activities (if the costs of producer services in road transport and moving activities increases by 1%, the price of bread and cereal products increases by 0.07%). In the case of the influence of all independent variables on the dependent variable, this is an inelastic change in the dependent variable (prices for bread and cereal products), as the calculated values do not exceed 1%.

The logarithmic model (LOG model) also proved to be the most suitable functional form in the model for the tertiary sector (distribution and trade). The coefficient of determination (R^2^) is 0.046, which means that the model explains only 4.6% of the variability of the dependent variable. The Durbin–Watson test value is 1.994, which indicates that there is no autocorrelation of the residual values (errors) in the regression model. The value of the F-statistic is 0.361, which is above the usual acceptable limit (0.05) for statistical significance or significance. The calculated elasticities for the individual independent variables show that positive changes (increases) in the prices of cereal products and the costs of advertising services and road freight transport influence the decline in the consumption of bread and cereal products. Based on the calculated elasticities for all other variables included (prices of imported and exported cereals and cereal products, labour costs in trade), it can be concluded that an increase in prices or costs in distribution and trade influences the increase in demand for bread and cereal products.

In this sector, three variables based on the calculated elasticities do not correspond to the theoretical assumption according to which the elasticities of the opposite values (+/−) should be calculated. These variables are the change in average import and export prices for bread and cereal products and the change in the costs of producer services in advertising. Due to the positive values of the calculated elasticities in both models, these three variables were excluded from the model (Table 10).

All three sectoral sub-models are combined here into a joint model, with which we have determined the influence (sensitivity test) of each sector on the retail price of bread and cereal products on the basis of the specified weights derived from the overall elasticities in the individual sectoral sub-models (Table 11).

### 3.2. Theoretical Model of Price Formation—Meat Sector

The model consists of sub-models for the primary, secondary and tertiary sectors. The purpose of each sectoral sub-model is to analyse the impact on the retail price of meat of the factors included that are specific to each sector of the agri-food chain for meat. In the model for the primary sector, which represents animal husbandry, we have included as important factors the labour costs in animal husbandry (gross wages in the agricultural activity of animal husbandry) and the evolution of the costs of agricultural inputs (energy and lubricants, feed, veterinary services, etc.). With these data, we have captured most of the costs typical of the primary sector of the meat agri-food chain. The sub-model for the secondary sector includes data on the evolution of prices for the slaughter animals of agricultural producers (which represent the starting price in the livestock sector), data on labour costs in the production of meat and meat products and data on the evolution of costs for raw materials, energy and transport. Based on a preliminary analysis of the functioning of the food processing sector in the meat chain, with a particular focus on the analysis of costs and value added, we can emphasise that we have covered most of the theoretically important factors that affect the level of the starting price or, due to the shifting of prices along the chain, also the retail price of meat. The sub-model for the tertiary sector, which includes distribution and trade activities, contains data on the change in prices of industrial products in food production (these are data on the initial price of the secondary sector, although this is not entirely accurate at this point, as it covers the whole food industry and not just the price of meat or meat products), data on labour costs in the wholesale and retail trade of meat and meat products, data on the change in the average import and export price of meat and meat products, and data on the change in the cost of advertising services and road freight transport. In each sectoral sub-model, we wanted to assess the impact on the retail price of meat of the factors associated with the activities of each sector, which is why we included data on the retail price of meat (consumer price index) as a dependent variable in all three models.

#### 3.2.1. Econometric Sub-Models for the Primary Sector (Combination of the PRICE/PRICE and CONSUMPTION/PRICE Models)

The properties of the econometric models we used to analyse the dependence of the change in the retail price of meat on the change in the wage level in the livestock industry and the change in the cost of agricultural inputs are presented. The functional forms of the econometric models (equations) are presented in Table 12.

All functional forms of the model are very similar according to the evaluation of the statistical and econometric tests. The average values of the coefficient of determination (R^2^) in all four functional forms of the model are around 0.093, which means that the independent variables together explain around 9% of the variability of the dependent variable *Y*. The value of the Durbin–Watson test for all functional forms of the model is around 1.839 and close to 2, which indicates that the model does not exhibit autocorrelation. The standard error varies depending on the functional form of the model and indicates how far the individual data are on average from the regression line. The F-statistic is statistically significant or significant (less than 0.05) in all functional forms of the model. The table of coefficients of the individual variables shows that only variable *X9* is statistically significant (its *p*-value is less than 0.05), and in addition, variables *X1*, *X2*, *X4* and *X5* are conditionally statistically significant (their *p*-value is between 0.051 and 0.350).

The calculations of the elasticities themselves are very similar in all four functional forms of the model. The elasticity calculations show (in the LOG-LIN form of the model) that a 1% increase in the average wage in the livestock sector and changes in the costs of seeds and seedlings, energy and lubricants, fertilisers and soil improvers, and feed and veterinary services do not significantly affect the change in meat prices. The same applies for changes in the cost of agricultural equipment (if the cost of agricultural equipment increases by 1%, the price of meat increases by 0.10%) and agricultural buildings (if the cost of agricultural buildings increases by 1%, the price of meat increases by 0.30%). If the cost for plant protection products, which are important for the production of animal feed and thus indirectly increase the cost for breeding, rises by 1, the price of meat falls by 0.10%. In the case of the influence of all independent variables on the dependent variable, this is an inelastic change in the dependent variable (meat prices), as the calculated values do not exceed 1%.

The dependent variable, which was represented in the base models by data on meat price, was replaced in all three models presented below by data on meat consumption, which was defined on the basis of accessible or available data on consumption. This replacement of the dependent variable results in a situation in which the values of the calculated elasticities for the individual variables should, at least theoretically, be the opposite of those of the base model (price/price model).

In the models for the primary sector (agricultural production sector), the log-linear form (LOG-LIN model) proved to be the most suitable functional form of the model, with a coefficient of determination (R^2^) of 0.024, which means that the model explains 2.4% of the variability of the variables. The value of the Durbin–Watson test is 2.10, which is close to 2 and means that there is no autocorrelation in the model. The value of the F-statistic is statistically insignificant or insignificant (greater than 0.05), which indicates that the regression model is statistically insignificant and that there is a relatively weak influence of an independent variable on the dependent variable. The calculated elasticities for the individual independent variables show that positive changes (increases) in the costs of energy and lubricants, fertilisers and means of soil improvement, and buildings influence the reduction in meat consumption. From the calculated elasticities for all other variables included, it can be concluded that the increase in prices or costs in agricultural production (animal husbandry) influences the increase in demand for meat.

A comparison of the values of the calculated elasticities in both models (PRICE/PRICE and CONSUMPTION/PRICE) for the variables included in the primary sector shows that two variables do not correspond to the theoretical assumption according to which the elasticities of the opposite values should be calculated (+/−) (Table 13). These two variables are the change in the cost of fertilisers and soil improvers and the change in the cost of agricultural equipment. In the case of the change in fertiliser and soil improver prices, the calculated elasticity in the PRICE/PRICE model was negative, which contradicts theoretical expectations and means that the retail price of bread and cereal products would fall if the cost of fertilisers and soil improvers increased. Despite the theoretically expected calculated elasticity in the CONSUMPTION/PRICE model, according to which an increase in the cost of fertilisers and soil improvers would reduce the demand for bread and cereal products, this variable was excluded from the final model. The same applies to the variable of cost development for agricultural equipment.

#### 3.2.2. Econometric Sub-Models for the Secondary Sector (Combination of the PRICE/PRICE and CONSUMPTION/PRICE Models)

We analysed the properties of the econometric models depending on the change in the retail price of meat as follows: changes in the prices of animals for slaughter at producers; changes in the level of wages in the production of meat and meat products; changes in raw material costs; changes in the costs of energy products; and changes in the value of the transport index. Functional forms of econometric models (equations) are presented in Table 14.

All four functional forms of the model are very similar in terms of the statistical and econometric test results. The values of the coefficient of determination (R^2^) are similar for all functional forms of the model, which means that the various transformations have not drastically improved the explanatory power of the model. The R^2^ values are around 0.128, which means that the independent variables together explain around 13% of the variability of the dependent variable *Y*. The average value of the Durbin–Watson test is around 1.792 for all functional forms of the model and is close to 2, which indicates that there is no autocorrelation. The standard error varies depending on the functional form of the model. The F-statistic is statistically significant in all functional forms of the model and is 0.001, which indicates that the regression model is statistically significant and that there is a relationship or correlation between the independent variable and the dependent variable. The table of coefficients of the individual variables (not shown here) indicates that variables *X3* and *X4* are statistically significant (their *p*-value is less than 0.05) or that they significantly influence the dependent variable *Y*. The variables *X1* and *X2* are also conditionally statistically significant, with the latter having a *p*-value greater than 0.05.

The calculation of the elasticities themselves does not differ significantly depending on the functional form of the model. The elasticity calculations show (in the LIN form of the model) that an increase in the average wage in the production of meat and meat products and the positive changes in the prices of agricultural products for producers by 1% influence the reduction in meat prices, which contradicts the expectations of the research. Changes in the cost of raw materials have a greater impact on the change in food price (if the cost of raw materials increases by 1%, the price of meat increases by 0.44%). Changes in the value of the transport index have a negligible impact on the change in the price of meat. In the case of the influence of all independent variables on the dependent variable, this is an inelastic change in the dependent variable (meat prices), as the calculated values do not exceed 1%.

We have chosen the logarithmic model (LOG model) as the most appropriate functional form of the model for the secondary sector (food industry sector). The coefficient of determination (R^2^) of this model is 0.031, which means that the model explains 3.1% of the variation in the dependent variable. The model has no autocorrelation, as the Durbin–Watson test value of 2.273 is relatively close to 2. The value of the F-statistic is 0.549 and is therefore above the normally acceptable limit (0.05) for statistical significance. The calculated elasticities for the individual independent variables show that positive changes (increases) in raw material cost and the transport index influence the decline in meat consumption. From the calculated elasticities for all other included variables (prices for slaughter animals at the producers, labour costs, energy costs), it can be concluded that the increase in prices or costs in the processing sector of the agri-food chain for meat influences the increase in demand for meat.

In this sector, the elasticities in both models (PRICE/PRICE and CONSUMPTION/PRICE) were calculated with opposite values for all five variables (Table 15), so that all five variables met the theoretical criteria and were included in the final model.

#### 3.2.3. Econometric Sub-Models for the Tertiary Sector (Combination of the PRICE/PRICE and CONSUMPTION/PRICE Models)

We used the properties of the econometric models to show the dependence of the change in the retail price of meat as follows: the change in wages in the wholesale trade of meat and meat products; changes in the level of retail wages in specialised shops selling meat and meat products; changes in the average import and export price of meat and meat products; changes in the prices of industrial products in food production; and changes in the cost of services in advertising and services in road freight transport and removal activities. The functional forms of the econometric models (equations) are presented in Table 16.

All four functional forms of the model are very similar in terms of the statistical and econometric test results. The coefficient of determination (R^2^) values are around 0.242, which means that the model explains about 24% of the variance of the dependent variable *Y*. The average value of the Durbin–Watson test for all functional forms of the model is around 2.105 and therefore close to 2, which indicates that there is no autocorrelation in the model. The standard error varies depending on the functional form of the model and indicates how far the individual data are on average from the regression line. The F-statistic is statistically significant or significant (less than 0.05) in all functional forms of the model. The table of coefficients of the individual variables (which is not shown here) shows that only variable *X5* is statistically significant (its *p*-value is less than 0.05) or that it significantly influences the dependent variable *Y*. In addition to variables *X1* and *X7*, which are conditionally statistically significant, all other variables are characterised by *p*-values greater than 0.05.

Elasticity calculations show (in the LIN form of the model) a minimal or negligible impact on the retail price of meat in the case of an increase in the average wage in wholesale and retail and in the case of changes in the average prices of imported and exported meat and meat products. If the average gross wage in the retail sector (retail in specialised shops with meat and meat products) and the average export price of meat and meat products increase by 1%, the price of meat increases by 0.01%. If the average gross wage in the wholesale trade (wholesale of meat and meat products) and the average import price of meat and meat products increase by 1%, the price of meat falls by 0.01%, which is somewhat contrary to the expectations of the research. Changes in the prices of industrial products at food manufacturers have a greater impact on the change in the price of meat (if the prices of industrial products at food manufacturers rise by 1%, the price of meat rises by 0.57%), on the cost of services for road haulage and moving activities for manufacturers (if the cost of services for road haulage and moving activities for manufacturers increases by 1%, the price of food increases by 0.09%) and on the cost of services for advertising for manufacturers (if the cost of services for advertising for manufacturers increases by 1%, the price of food decreases by 0.04%). In the case of the influence of all independent variables on the dependent variable, this is an inelastic change in the dependent variable (meat prices), as the calculated values do not exceed 1%.

The logarithmic model (LOG model) also proved to be the most suitable functional form in the model for the tertiary sector (distribution and trade). The coefficient of determination (R^2^) is 0.045, which means that the model explains only 4.5% of the variability of the dependent variable. There is no autocorrelation in the model as the Durbin–Watson test value is close to 2, exactly 2.157. The value of the F-statistic is 0.414, which is above the usual acceptable limit (0.05) for statistical significance or meaningfulness. The calculated elasticities for the individual independent variables show that positive changes (increases) in the prices of imported and exported meat and meat products as well as the costs of advertising services and road transport influence the decline in meat consumption. From the elasticities calculated for all other variables included, it can be concluded that the increase in prices or costs in the areas of distribution and trade influences the increase in demand for meat.

In this sector, four variables based on the calculated elasticities do not correspond to the theoretical assumption according to which the elasticities of the opposite values (+/−) should be calculated (Table 17). These variables are as follows: changes in average import prices for meat, changes in retail wage levels in speciality shops selling meat and meat products, changes in the prices or costs of industrial products at manufacturers in food production, and changes in the costs of advertising services. Due to the values of the calculated elasticities with the same sign in both models, these four variables were excluded from the final model.

All three sectoral sub-models are combined here into a joint model, with which we have determined the influence (sensitivity test) of each sector on the retail price of meat on the basis of the specified weights derived from the overall elasticities in the individual sectoral sub-models (Table 18).

### 3.3. Theoretical Model of Price Formation—Dairy Sector

The model consists of three sectoral sub-models that represent the three basic sectors of the food chain with milk and egg production. These sectoral sub-models are used to analyse the impact of individual factors that are reflected in the retail prices of milk, cheese and eggs in all three sectors. In the sub-model for the primary sector, which represents the agricultural activity of milk processing, we have the labour costs in milk processing (gross wages in milk processing) and the changes in agricultural input costs (energy and lubricants, feed, veterinary services, etc.). With these data, we have captured most of the typical costs in the agri-food chain for the production of milk and eggs. The sub-model for the secondary sector includes data on the evolution of cow’s milk prices among agricultural producers (which represent the starting price in the milk processing sector), data on labour costs in milk processing and data on the evolution of costs for raw materials, energy products and the transport index. Based on a preliminary analysis of the factors or identified costs typical of the dairy processing food industry sector, we can emphasise that we have captured most of the more important factors affecting the value added of agricultural and food products in this sector. The sub-model for the tertiary sector, which includes distribution and trade activities, contains data on the change in prices of manufactured food products (i.e., data on the change in prices of all manufactured food products together and not just data on the change in prices of manufactured dairy products), data on the labour force in the wholesale and retail trade of milk and dairy products and eggs, data on the change in the average import and export price of dairy products and eggs, and data on the change in costs of services such as advertising and road freight transport. In all three sectoral sub-models, we analysed the influence of these factors on the retail price of milk, cheese and eggs, using data on the retail price index for milk, cheese and eggs (consumer price index) as the dependent variable in the models.

#### 3.3.1. Econometric Sub-Models for the Primary Sector (Combination of the PRICE/PRICE and CONSUMPTION/PRICE Models)

The properties of the econometric models that we used to analyse the dependence of the change in the retail price of milk, cheese and eggs on the change in the wages earned in milk production and the change in the costs of agricultural inputs are presented. The functional forms of the econometric models are presented in Table 19.

All four functional forms of the model are very similar in terms of the statistical and econometric test results. The coefficient of determination (R^2^) values are about 0.096, which means that the independent variables together explain about 9.6% of the variability of the dependent variable *Y*. The average value of the Durbin–Watson test is around 1.901 for all functional forms of the model and is close to 2, which indicates that there is no autocorrelation in the model. The standard error varies depending on the functional form of the model and indicates how far the individual data are on average from the regression line. The F-statistic is statistically significant for all functional forms of the model (less than 0.05). The table of coefficients of the individual variables (which is not shown here) shows that no variable is statistically significant (its *p*-value is greater than 0.05), but the variables *X1*, *X2*, *X5* and *X7* are conditionally statistically significant (their *p*-value is between 0.051 and 0.150).

The calculation of the elasticities themselves does not differ significantly depending on the functional form of the model. The elasticity calculations show (in the LOG-LIN form of the model, which we have chosen as the most appropriate) that a 1% increase in the average wage in dairy farming and a positive change in the costs of energy and lubricants, fertilisers and soil improvers, animal feed, and buildings do not significantly affect the changes in the price of milk, cheese and eggs. Changes in the cost of seeds and seedlings (if the cost of seeds and seedlings increases by 1, the price of milk, cheese and eggs increases by 0.10), crop protection products (if the cost of crop protection products increases by 1%, the price of milk, cheese and eggs increases by 0.10%), veterinary services (if the cost of crop protection products increases by 1%, the price of milk, cheese and eggs increases by 0.10%) and agricultural equipment (if the cost of agricultural equipment increases by 1%, the price of milk, cheese and eggs increases by 0.10%) have a greater impact on the price of milk, cheese and eggs. In the case of the influence of all independent variables on the dependent variable, this is an inelastic change in the dependent variable (prices for milk, cheese and eggs), as the calculated values do not exceed 1%.

In all three models presented below, the dependent variable was replaced, while all independent variables remained unchanged. The dependent variable, which in the basic models was represented by data on the price development of milk, cheese and eggs, was replaced by data on the consumption of milk, cheese and eggs, which we defined ourselves using the available statistical data. With this replacement of the dependent variable, we achieved a situation in which the values of the calculated elasticities for the individual variables should at least theoretically be the opposite of those in the base model.

As the most appropriate functional form of the model for the primary sector (agricultural production sector), we have chosen a log-linear model (LIN-LOG model) with a coefficient of determination (R^2^) of 0.025, which means that the model explains only 2.5% of the variability of the dependent variable. The model has no autocorrelation as the Durbin–Watson test value is close to 2, exactly 2.041. The value of the F-statistic is statistically insignificant or insignificant (greater than 0.05), indicating that the regression model is statistically insignificant and that there is a relatively weak influence of an independent variable on the dependent variable. The calculated elasticities for the individual independent variables show that positive changes (increases) in the wage bill in milk production, the costs of plant protection products, the costs of animal feed, the costs of veterinary services, the costs of agricultural equipment and the costs of agricultural buildings influence the decrease in the consumption of milk, cheese and eggs. Based on the calculated elasticities for all other variables included, we can conclude that the increase in prices or costs in agricultural production (milk production) influences the increase in demand for milk, cheese and eggs.

In the sub-model for the primary sector, there are two variables that do not correspond to the theoretical research expectations according to which the elasticities of the opposite values (+/−) should be calculated. At this point, the values of the calculated elasticities for the variables of changes in the costs of seeds and seedlings and changes in the costs of fertilisers and soil improvers in the PRICE/PRICE model were positive, which was theoretically expected. This means that with the increase in the cost of seeds and seedlings, as well as the cost of fertilisers and soil improvers, the retail price of milk, cheese and eggs would also increase. However, due to the positive value of the calculated elasticities in the CONSUMPTION/PRICE model, these two variables were excluded from the common price construction model, which is contrary to the theoretical expectations according to which the calculated elasticities of the opposite values (+/−) should have been (Table 20).

#### 3.3.2. Econometric Sub-Models for the Secondary Sector (Combination of the PRICE/PRICE and CONSUMPTION/PRICE Models)

The properties of the econometric models we used to analyse the dependence of the change in the retail price of milk, cheese and eggs on the following factors are presented: changes in cow’s milk prices among producers; wage changes in milk processing; changes in raw material costs; changes in energy costs; and changes in the transport index. The functional forms of the econometric models are presented in Table 21.

All functional forms of the model are very similar in terms of the statistical and econometric test results. The coefficient of determination (R^2^) values are around 0.324, which means that the models can explain about 32% of the variability of the dependent variable. The average value of the Durbin–Watson test for all functional forms of the model is approximately 1.861 and is close to 2, indicating that there is no autocorrelation in the model. The standard error varies depending on the functional form of the model. The F-statistic is statistically significant (*p* < 0.050), indicating that the regression model explains a significant proportion of the variance in the dependent variable. This suggests there is a relationship between the independent variables and the dependent variable. The table of coefficients of the individual variables (which is not shown here) indicates that only one variable (*X4*) is statistically significant (its *p*-value is less than 0.05) or that it significantly influences the dependent variable *Y*. The other variables have *p*-values of less than 0.05, indicating that the regression model is statistically significant. The other variables have *p*-values above 0.05, which means that they are not statistically significant. In the model with the functional form LOG and LIN-LOG, the variable *X1* is also conditionally statistically significant (its *p*-value is between 0.051 and 0.350).

Elasticity calculations show (in the LIN form of the model) that changes in the one increase in the average wage in milk processing and in commodity prices do not significantly affect changes in the prices of milk, cheese and eggs. A change in the cost of energy products has a slightly greater impact on the price of milk, cheese and eggs (if the cost of electricity, gas and other fuels increases by 1%, the price of milk, cheese and eggs increases by 0.01%), on the value of the transport index (if the value of the transport index increases by 1%, the price of milk, cheese and eggs increases by 0.02%) and on the producer milk price (if the producer milk price increases by 1%, the price of milk, cheese and eggs increases by 0.02%). In the case of the influence of all independent variables on the dependent variable, this is an inelastic change in the dependent variable (prices for milk, cheese and eggs), as the calculated values do not exceed 1%.

We have chosen the linear-logarithmic model (LIN-LOG model) as the most appropriate functional form of the model for the secondary sector (food industry sector). The coefficient of determination (R^2^) in this model is 0.03, which means that the model explains 3% of the variation in the dependent variable. There is no autocorrelation in the model, as the value of the Durbin–Watson test is 2.046, which is very close to the desired value of 2. The value of the F-statistic is 0.523 and is therefore above the usually acceptable limit (0.05) for statistical significance. The calculated elasticities for the individual independent variables show that positive changes (increases) in the level of labour costs in milk processing, transport index values and energy costs influence the decrease in the consumption of milk, cheese and eggs. From the calculated elasticities for all other included variables (cow’s milk prices of producers, raw material costs), it can be concluded that the increase in prices or costs in the processing sector of the agri-food chain with milk production influences the increase in demand for milk, cheese and eggs.

In this sector, the value of the calculated elasticity for the variable of the change in the price of cow’s milk among producers in the PRICE/PRICE model was positive, which is theoretically to be expected and means that if the price of cow’s milk among producers increases, the retail price of milk, cheese and eggs would also increase. However, due to the positive value of the calculated elasticity in the CONSUMPTION/PRICE model, this variable was excluded from the joint pricing model, as the results of the calculated elasticities did not match the theoretical assumptions according to which the elasticities of the opposite values (+/−) should be calculated (Table 22).

#### 3.3.3. Econometric Sub-Models for the Tertiary Sector (Combination of the PRICE/PRICE and CONSUMPTION/PRICE Models)

This page presents the properties of the econometric models we used to analyse the dependence of the change in the retail price of milk, cheese and eggs on the following factors: changes in the level of wages in the wholesale trade of milk, dairy products, eggs, edible oils and fats; changes in the level of wages in the retail trade in specialised shops of food, beverages and tobacco products; changes in the average import and export price of dairy products and eggs; changes in the prices of industrial products in food production; and changes in the costs of services in advertising and services in road freight transport and moving activities. The functional forms of the econometric models are presented in Table 23.

All functional forms of the model are very similar in terms of the statistical and econometric test results. The coefficient of determination (R^2^) values are around 0.290, which means that the model explains around 29% of the variance of the dependent variable *Y*. The average value of the Durbin–Watson test is around 2.256 for all functional forms of the model and is close to 2, which indicates that there is no autocorrelation in the model. The standard error varies depending on the functional form of the model and indicates how far the individual data are on average from the regression line. The F-statistic is statistically significant or significant (less than 0.05) in all functional forms of the model. The table of coefficients of the individual variables (not shown here) shows that only the variable *X5* is statistically significant (its *p*-value is less than 0.05) or has a significant influence on the dependent variable *Y*. However, other variables have *p*-values higher than 0.05, while the variables *X1*, *X2*, *X6* and *X7* are conditionally statistically significant (their *p*-value is between 0.051 and 0.350).

Elasticity calculations show (in the LOG-LIN form of the model) that changes in the average price of imported and exported dairy products and eggs (price increase) of 1% have no effect on changes in the prices of milk, cheese and eggs. A minimal or negligible impact is seen in the increase in the average wage in wholesale (wholesale of milk, dairy products, eggs, edible oils and fats) and retail (retail in specialised food, beverages and tobacco shops). If the average wage in wholesale increases by 1%, the price of milk, cheese and eggs increases by 0.01%. If the average wage in the retail sector increases by 1%, the price of milk, cheese and eggs falls by 0.01%, which somewhat contradicts the expectations of the research.

Changes in the prices of manufactured products at food manufacturers have a greater impact on the price of milk, cheese and eggs (if the prices of manufactured products at food manufacturers increase by 1%, the price of milk, cheese and eggs increases by 0.90%), on the costs of producer services in advertising (if the costs of producer services in advertising increase by 1%, the price of milk, cheese and eggs increases by 0.10%) and the cost of producer services in road transport and moving activities (if the cost of producer services in road transport and moving activities increases by 1%, the price of milk, cheese and eggs decreases by 0.10%, which to some extent contradicts the predictions of the study). In the case of the influence of all independent variables on the dependent variable, this is an inelastic change in the dependent variable (prices for milk, cheese and eggs), as the calculated values do not exceed 1%.

In the model for the tertiary sector (distribution and trade), however, the logarithmic form (LOG model) proved to be the most suitable functional form. The coefficient of determination (R^2^) is 0.046, which means that the model explains only 4.6% of the variability of the dependent variable. The value of the Durbin–Watson test is very close to 2, namely exactly 1.994, which indicates that there is no autocorrelation in the regression model. The value of the F-statistic is 0.469, which is above the usual acceptable limit (0.05) for statistical significance. The calculated elasticities for the individual independent variables show that positive changes (increases) in retail labour costs, changes in the prices of imported dairy products and eggs, the prices of industrial dairy products and the costs of advertising services and road freight transport influence the decline in the consumption of milk, cheese and eggs. Based on the calculated elasticities for all other included variables (prices of exported dairy products and eggs, labour costs in wholesale milk, dairy products and eggs), we can conclude that an increase in prices or costs in distribution and trade activities causes an increase in demand for milk, cheese and eggs.

In this sector, three variables based on the calculated elasticities do not correspond to the theoretical assumption according to which the elasticities of the opposite values (+/−) should be calculated. These variables are changes in wage levels in wholesale and retail trade and changes in the costs of road freight transport and relocation activities. Due to the values of the calculated elasticities with the same sign in both models (PRICE/PRICE and CONSUMPTION/PRICE), these three variables were excluded from the final model (Table 24).

All three sectoral sub-models are combined here into a joint model with which we have determined the influence of the individual sectors on the retail price of milk, cheese and eggs on the basis of the specified weights derived from the overall elasticities in the individual sectoral sub-models (Table 25).

## 4. Discussion

Differences in the calculations of the individual agri-food systems, which show the flexibility or sensitivity of the individual subsystems, can be attributed to various reasons, which are due to the complexity of understanding agri-food systems, especially in the Slovenian area, which is specific compared to the agri-food systems of the other EU countries. This is mainly due to its small size (mostly short- and medium-length market channels), the diversity of the geographical structure, the specificity of the connection of actors between sectors and the general knowledge of the functioning of agri-food systems and accessible databases.

The results show that the agricultural and food systems analysed in Slovenia exhibit a similar distribution of the sensitivity or dynamics of changes in the subsectors in relation to the changes in costs (sector 1 > sector 3 > sector 2). Similar results can be seen in the common “food” sector, which can be compared with the data from the EU member states. The clearest differences between the subsectors occur in the dairy sector, which is partly due to the standardised collection of data for the milk, cheese and eggs group, of which subsector 2 may represent different complex processing methods and associated costs. Similar results are also observed in the bread and cereal product sector, with relatively equal sensitivity between subsector 1 and subsector 3. The understanding and knowledge of cost trends at the production stage varies from year to year, with the level of co-operation and links between actors in subsectors 1 and 3 being relatively dynamic each year. Multi-year representations of purchase prices of agricultural and food products from primary producers in the meat and dairy sectors are consistent with the calculations of the relatively small share of subsector 1 compared to the bread and cereal product sector. A common finding for all agri-food systems is that the sensitivity of all subsectors is low, with the exception of subsector 3 in the dairy agri-food chain. However, the overall assessment shows a dynamic level of sensitivity in all sectoral areas.

The statistical data obtained on the fluctuations in the purchase prices of meat, cereals and milk over a long period between 2010 and 2023 rightly show the results of the sensitivity expressed in % in subsector 1. In the case of cereals, 11 major deviations were observed (these are isolated deviations showing a deviation between 20% and 30%)—sensitivity, 49.9%; in the dairy sector, there were 4 such major deviations—sensitivity, 17.6%; and in the case of meat sector 2 (excluding the pork sector, where there were 6)—sensitivity, 20.2%. It is useful to combine these data with sensitivity calculations of individual subsectors within individual food chains and the distribution of the share of sensitivity along the chain. Differences in the sensitivity of subsector 3 are particularly evident in milk and dairy products (a deviation of around 30%), while it is roughly the same in the meat and cereal chains. This can be attributed to the need for greater flexibility and adaptation to the higher labour costs associated with covering a larger group of milk and dairy products than in the other two food chains.

The individual data show that the calculated cost elasticities are sometimes exaggerated or overestimated, which we attribute to the data basis and the lack of a breakdown to the appropriate levels that would show a direct impact on the individual subsector. Based on the report and the results of the project, we can present some guidelines and recommendations that we believe could help, as we wrote in the introduction, to understand the behaviour and response of agricultural and food systems to certain cost changes:

(1) Updating databases that would help to understand the distribution of costs in the cereal and dairy chain (mainly in the processing industry phase).
(2) Creation of centralised databases for the collection of non-confidential data that would contribute to a comprehensive understanding of the functioning of agri-food systems. Information on prices, costs and profit margins throughout the value chain can help to better and more quickly identify market failures. Information on the determinants of prices, costs and margins can help develop policies to address market failures and increase competitiveness.
(3) Regular monitoring of prices at the level of the individual agricultural and food sectors. The shorter the information delay and the more detailed the information, the faster governments receive a signal about disruptions that negatively affect the efficient market balance in a specific market. Furthermore, policy makers can intervene in inefficient markets by providing value chain actors with market information that is otherwise difficult to obtain.
(4) After further analyses, the results obtained could lead to the development of mitigation measures in case of major changes at the level of individual agricultural and food sectors.

The methodological approach used in this study is similar to that described as in study [13] and is based on standard economic models. The authors used an example of the logarithmic form of the functions using the Cobb–Douglas production function to estimate the pricing of products (not just food). Such a form of the function is appropriate, but we believe that it is not precise enough to analyse the construction of product prices in food chains in the way presented in our research. The agri-food sector is specific from this point of view as it requires micro-level analysis, e.g., when input costs are repeated in different sectors and cannot be fully separated. The logarithmic form is therefore appropriate, but with the specification of newly created variables to reflect the impact on the change in food prices. In the study [22], the authors estimated a linear structural vector autoregression (SVAR) model to examine the impact of nominal gasoline price shocks on food prices. Their results show that the effect of an unexpected increase in the nominal gasoline price significantly increases the response of aggregate food prices five months after the shock. The research is precise and focuses mainly on analysing the impact of fuel price changes, which does not take into account the fact that such costs are incurred in different sectors along the agricultural and food chains. We studied such and other research before deciding to develop this complex modelling system to comprehensively analyse food prices, which is presented in this article. In addition, the model also proved useful at the micro level, as it assesses the sensitivity of individual food chains. As for the theoretical and practical consequences of the results presented, it is difficult to derive direct facts and considerations on food markets, as the model presented is primarily intended as a supporting tool for analysis in the agricultural profession and indirectly in politics. Limitations and thus opportunities for further development of the research can be seen above all in the need for an improvement of the collection of input data for the analysis, greater transparency of the data in relation to the sectoral system and a detailed analysis of individual agricultural and food chains. In our opinion, the additional methodological econometric approaches are sufficiently well known, useful and diverse and could be used for further analyses of this kind in the future (such as testing VAR model).

## 5. Conclusions

The article presents the construction of a theoretical model for the formation of the price of production products. The results of the research project on the federal economy also connect the lower part of the costs with the overall analysis of the world of these relevant sectors, and they are directly skewed. We find that the dispersion of sensitivity and intangibility in the market is not uniform across sectors. This can be attributed to the greater movement of information through the agri-food chains. Each actor in the value chain has a higher priority in terms of the type of information obtained and from whom. Value chain actors, such as primary producers, processors and retailers, have mediocre detailed information, prices and/or have nomadic habits. In the context of the world’s politics, market information is common for the common system of market efficiency. Information about prices, costs and margins in the value chain can be better and more easily signalled by market failures. Information about the factors that determine prices, baskets of goods and margins is one level in the cancellation of policy aimed at eliminating market objectivities and reducing incidence. The smaller the information gap and the more detailed the information, the faster governments receive a signal about disturbances that affect the efficient market equilibrium in a specific market. Furthermore, policy makers can intervene in inefficient markets by providing value chain actors with market information that is otherwise difficult to obtain.

With the results presented, we have probably succeeded in contributing to the understanding of the functioning and responsiveness of agri-food systems and in providing the basis for preventive measures in the area of stabilising market conditions in the agri-food sector. This study was conducted with the example of Slovenia, and we believe that the methodology presented is suitable for an even more comprehensive and in-depth analysis.

## Figures and Tables

**Table 1:** Basic differences between the VAR model and standard forms of econometric models (according to [33,34]).

Scenario	VAR Models	Standard Econometric Models
Purpose	To model and analyse dynamic, interdependent relationships among multiple variables.	To estimate causal relationships or quantify the effect of independent variables on a dependent variable.
Dynamic Systems	Use when variables are interdependent and influence each other over time.	Use when the goal is to estimate the effect of specific variables on an outcome.
Forecasting	Well suited for multivariate time series forecasting.	Use simpler time series models for univariate forecasting.
Causal Analysis	Not ideal for causal inference.	Best for testing hypotheses about causal relationships.
Focus	Dynamic interrelationships and feedback effects.	Elasticity and proportional relationships.
Outputs	Coefficients, impulse response functions (IRFs), forecast error variance decomposition.	Elasticities of the dependent variable with respect to independent variables.
Examples	Economic indicators (GDP, inflation, interest rates).	Demand analysis, cost functions, production functions.

**Table 2:** Results of the ADF test.

Results of Adf Test—Example of Cereal Sector				
	ADF Statistic	*p*-Value	Number of Observations Used	Critical Values
Primary Sector				
*Y*	−4.635	0.000	210	1%: −3.461878735881654 5%: −2.875403665910809 10%: −2.574159410430839
*X1*	−0.476	0.897	201	1%: −3.4633090972761744 5%: −2.876029332045744 10%: −2.5744932593252643
*X2*	−5.556	0.000	212	1%: −3.4615775784078466 5%: −2.875271898983725 10%: −2.5740891037735847
*X3*	−6.704	0.000	211	1%: −3.46172743446274 5%: −2.8753374677799957 10%: −2.574124089081557
*X4*	−9.894	0.000	215	1%: −3.461136478222043 5%: −2.875078880098608 10%: −2.5739861168199027
*X5*	−13.930	0.000	215	1%: −3.461136478222043 5%: −2.875078880098608 10%: −2.5739861168199027
*X6*	−4.042	0.001	211	1%: −3.46172743446274 5%: −2.8753374677799957 10%: −2.574124089081557
*X7*	−15.063	0.000	215	1%: −3.461136478222043 5%: −2.875078880098608 10%: −2.5739861168199027
*X8*	−3.109	0.026	202	1%: −3.4631437906252636 5%: −2.8759570379821047 10%: −2.574454682874228
Secondary sector				
*Y*	−3.088	0.027	152	1%: −3.474120870218417 5%: −2.880749791423677 10%: −2.5770126333102494
*X1*	−10.527	0.000	147	1%: −3.4756368462466662 5%: −2.8814104466172608 10%: −2.5773652982553568
*X2*	−0.705	0.846	143	1%: −3.4769274060112707 5%: −2.8819726324025625 10%: −2.577665408088415
*X3*	−4.111	0.001	155	1%: −3.4732590518613002 5%: −2.880374082105334 10%: −2.5768120811654525
*X4*	−2.578	0.098	143	1%: −3.4769274060112707 5%: −2.8819726324025625 10%: −2.577665408088415
*X5*	−11.574	0.000	154	1%: −3.473542528196209 5%: −2.880497674144038 10%: −2.576878053634677
Tertiary sector				
*Y*	−6.294	3.533 ×10^−8^	185	1%: −3.4662005731940853 5%: −2.8772932777920364 10%: −2.575167750182615
*X1*	−1.182	0.681	175	1%: −3.4682803641749267 5%: −2.8782017240816327 10%: −2.5756525795918366
*X2*	0.610	0.988	176	1%: −3.4680615871598537 5%: −2.8781061899535128 10%: −2.5756015922004134
*X3*	−4.030	0.001	176	1%: −3.4680615871598537 5%: −2.8781061899535128 10%: −2.5756015922004134
*X4*	−6.051	1.275 × 10^−7^	176	1%: −3.4680615871598537 5%: −2.8781061899535128 10%: −2.5756015922004134
*X5*	−4.378	0.000	187	1%: −3.465811691080702 5%: −2.877123351472649 10%: −2.5750770662586864
*X6*	−2.793	0.059	179	1%: −3.4674201432469816 5%: −2.877826051844538 10%: −2.575452082332012
*X7*	−1.292	0.633	173	1%: −3.4687256239864017 5%: −2.8783961376954363 10%: −2.57575634100705

**Table 3:** Results of the DW test.

Sector/Type of the Model	PRICE/PRICE	CONSUMPTION/PRICE
Cereal primary sector	LIN—2.34	LOG—1.97
Cereal secondary sector	LIN—2.17	LIN-LOG—2.08
Cereal tertiary sector	LIN—2.27	LOG—1.99
Meat primary sector	LOG-LIN—1.84	LOG-LIN—2.10
Meat secondary sector	LIN—1.79	LOG—2.27
Meat tertiary sector	LIN—2.10	LOG—2.15
Dairy primary sector	LOG-LIN—1.90	LIN-LOG—2.04
Dairy secondary sector	LIN—1.89	LIN-LOG—2.04
Dairy tertiary sector	LOG-LIN—2.25	LOG—1.98

**Table 4:** Reported values of the VIF test for selected models within individual agri-food chains.

	PRICE/PRICE MODELS								
Independent Variable	Cereal Primary Sector	Cereal Secondary Sector	Cereal Tertiary Sector	Meat Primary Sector	Meat Secondary Sector	Meat Tertiary Sector	Dairy Primary Sector	Dairy Secondary Sector	Dairy Tertiary Sector
*X1*	1.074	1.048	2.531	1.121	1.237	7.537	1.179	1.255	4.729
*X2*	1.431	2.786	3.110	1.437	2.413	7.195	1.438	2.391	5.186
*X3*	1.095	1.165	1.077	1.082	1.388	1.109	1.108	1.240	1.070
*X4*	1.128	2.803	1.050	1.129	2.498	1.094	1.129	2.753	1.114
*X5*	1.114	1.093	1.674	1.193	1.045	1.614	1.194	1.048	1.650
*X6*	1.221		1.064	1.203		1.033	1.209		1.032
*X7*	1.366		1.954	1.177		1.824	1.196		1.820
*X8*	1.205			1.442			1.470		
*X9*				1.238			1.253		
	**CONSUMPTION/PRICE MODELS**								
**Independent Variable**	**Cereal Primary** **Sector**	**Cereal Secondary** **Sector**	**Cereal Tertiary** **Sector**	**Meat Primary** **Sector**	**Meat Secondary** **Sector**	**Meat Tertiary Sector**	**Dairy Primary Sector**	**Dairy Secondary Sector**	**Dairy Tertiary Sector**
*X1*	1.049	1.034	2.590	1.152	1.299	6.594	1.216	1.068	3.905
*X2*	1.428	2.479	3,110	1.439	2.174	6.233	1.395	1.549	3.928
*X3*	1.109	1.157	1.058	1.084	1.409	1.139	1.127	1.113	1.107
*X4*	1.134	2.468	1.048	1.285	2.322	1.136	1.103	1.524	1.127
*X5*	1.111	1.073	1.689	1.221	1.032	1.780	1.228	1.066	1.121
*X6*	1.233		1.045	1.283		1.064	1.201		1.039
*X7*	1.369		2.034	1.181		1.901	1.260		1.037
*X8*	1.226			1.514			1.404		
*X9*				1.361			1.177		

**Table 5:** Equations of functional forms of econometric models PRICE/PRICE (BREAD AND GRAIN PRODUCTS—primary sector).

Type of the Formulation	Equations
LIN	*Yb* = 77.433 + 0.00002172 * *X1b* + 0.198 * *X2* − 0.008 * *X3* − 0.002 * *X4* − 0.163 * *X5* + 0.139 * *X6* − 0.129 * *X7* + 0.193 * *X8*
LOG-LIN	LNYb = 4.384 + 4.403 × 10^−7^ * *X1b* + 0.002 * *X2* − 0.00008049 * *X3* − 0.00001408 * *X4* − 0.002 * *X5* + 0.001 * *X6* − 0.001 * *X7* + 0.002 * *X8*
LIN-LOG	*Yb* = −3.612 − 0.061 * ln*X1b* + 20.475 * ln*X2* − 1.005 * ln*X3* − 0.084 * ln*X4* – 16.303 * ln*X5* + 13.318 * ln*X6* – 13.788 * ln*X7* + 20.026 * ln*X8*
LOG	LNYb = 3.588 + 0 * ln*X1b* + 0.194 * ln*X2* − 0.01 * ln*X3* + 0 * ln*X4* − 0.161 * ln*X5* + 0.127 * ln*X6* − 0.126 * ln*X7* + 0.198 * ln*X8*

**Table 6:** Comparison of the calculated elasticities or determination of the opposite elasticities in the econometric models PRICE/PRICE and CONSUMPTION/PRICE (BREAD AND CEREAL PRODUCTS—primary sector).

Elasticities in the PRICE/PRICE Model (Primary Sector)									
	*X1*	*X2*	*X3*	*X4*	*X5*	*X6*	*X7*	*X8*	*X9*
**LIN**	0.00	0.20	−0.01	0.00	−0.16	0.14	−0.13	0.19	/
**Elasticities of the CONSUMPTION/PRICE Model (Primary Sector)**									
	*X1*	*X2*	*X3*	*X4*	*X5*	*X6*	*X7*	*X8*	*X9*
**LOG**	0.07	1.06	4.69	−3.34	7.57	−1.03	7.32	−14.14	/

**Table 7:** Equations of functional forms of econometric models PRICE/PRICE (BREAD AND CEREAL PRODUCTS—secondary sector).

Type of the Formulation	Equations
LIN	*Yb* = 74.66 − 0.01 * *X1b* + 0.0 * *X2b* + 0.261 * *X3* + 0.176 * *X4* − 0.004 * *X5*
LOG-LIN	LNYb = 4.354 − 0.00009806 * *X1b* − 0.000004145 * *X2b* + 0.003 * *X3* + 0.002 * *X4* − 0.0000416 * *X5*
LIN-LOG	*Yb* = − 15.191 − 1.088 * ln*X1b* − 0.265 * ln*X2b* + 26,267 * ln*X3* + 1.298 * ln*X4* − 0.291 * ln*X5*
LOG	LNYb = 3.463 − 0.011 * ln*X1b* − 0.003 * ln*X2b* + 0.26 * ln*X3* + 0.013 * ln*X4* − 0.003 * ln*X5*

**Table 8:** Comparison of the calculated elasticities or determination of the opposite elasticities in the econometric models PRICE/PRICE and CONSUMPTION/PRICE (BREAD AND CEREAL PRODUCTS—secondary sector).

Elasticities in the PRICE/PRICE Model (Secondary Sector)					
	*X1*	*X2*	*X3*	*X4*	*X5*
**LIN**	−0.01	0.00	0.26	0.01	0.00
**Elasticities of the CONSUMPTION/PRICE Model (Secondary Sector)**					
	*X1*	*X2*	*X3*	*X4*	*X5*
**LIN-LOG**	3.02	−5.33	293.44	−3.28	−5.88

**Table 9:** Equations of functional forms of econometric models PRICE/PRICE (BREAD AND CEREAL PRODUCTS—tertiary sector).

Type of the Formulation	Equations
LIN	*Yb* = 48.445 + 0.0 * *X1b* + 0.00001864 * *X2b* + 0.008 * *X3b* + 0.007 * *X4b* + 0.509 * *X5* − 0.073 * *X6* + 0.069 * *X7*
LOG-LIN	LNYb = 4.112 − 0.000002909 * *X1b* + 5,334E−07 * *X2b* + 0.00007896 * *X3b* + 0.00007673 * *X4b* + 0.005 * *X5* − 0.001 * *X6* + 0.001 * *X7*
LIN-LOG	*Yb* = − 152.208 − 0.634 * ln*X1b* − 0.059 * ln*X2b* + 0.614 * ln*X3b* + 0.631 * ln*X4b* + 53,903 * ln*X5* – 5.967 * ln*X6* + 6.725 * ln*X7*
LOG	LNYb = 2.182 − 0.006 * ln*X1b* + 0.0 * ln*X2b* + 0.006 * ln*X3b* + 0.007 * ln*X4b* + 0.51 * ln*X5* − 0.058 * ln*X6* + 0.072 * ln*X7*

**Table 10:** Comparison of the calculated elasticities and determination of the opposite elasticities in the econometric models PRICE/PRICE and CONSUMPTION/PRICE (BREAD AND CEREAL PRODUCTS—tertiary sector).

Elasticities in the PRICE/PRICE Model (Tertiary Sector)							
	*X1*	*X2*	*X3*	*X4*	*X5*	*X6*	*X7*
**LIN**	0.00	0.00	0.01	0.01	0.51	−0.07	0.07
**Elasticities of the CONSUMPTION/PRICE Model (Tertiary Sector)**							
	*X1*	*X2*	*X3*	*X4*	*X5*	*X6*	*X7*
**LOG**	0.17	0.20	0.68	0.03	−4,36	−10.73	−11.66

**Table 11:** Maximum sensitivity to price changes for agricultural and food products in the event of changes in the cost level within each sector in the BREAD AND CEREAL PRODUCTS model.

Maximum Sensitivity for Price Changes in Agricultural Products in the Event of Cost Changes in a Single Sector (Calculated Weights—in %)		
Primary Sector	Secondary Sector	Tertiary Sector
51.2	2.2	46.6

**Table 12:** Equations of functional forms of econometric models PRICE/PRICE (meat—primary sector).

Type of the Formulation	Equations
LIN	*Yc* = 63.953 + 0.001 * *X1c* + 0.044 * *X2* + 0.006 * *X3* − 0.018 * *X4* − 0.114 * *X5* + 0.013 * *X6* + 0.032 * *X7* + 0.097 * *X8* + 0.295 * *X9*
LOG-LIN	LNYc = 4.252 + 0.000005029 * *X1c* + 0.0 * *X2* + 0.00006107 * *X3* + 0.0 * *X4* − 0.001 * *X5* − 0.0 * *X6* + 0.0 * *X7* + 0.001 * *X8* + 0.003 * *X9*
LIN-LOG	*Yc* = − 68.485 + 0.603 * ln*X1c* + 4,436 * ln*X2* + 0.522 * ln*X3* − 1.565 * ln*X4* − 11.767 * ln*X5* + 1.27 * ln*X6* + 3.421 * ln*X7* + 10.095 * ln*X8* + 29,288 * ln*X9*
LOG	LNYc = 2.955 + 0.006 * ln*X1c* + 0.045 * ln*X2* + 0.005 * ln*X3* − 0.016 * ln*X4* − 0.116 * ln*X5* + 0.012 * ln*X6* + 0.034 * ln*X7* + 0.098 * ln*X8* + 0.287 * ln*X9*

**Table 13:** Comparison of the calculated elasticities and determination of the opposite elasticities in the econometric models PRICE/PRICE and CONSUMPTION/PRICE (meat—primary sector).

Elasticities in the PRICE/PRICE Model (Primary Sector)									
	*X1*	*X2*	*X3*	*X4*	*X5*	*X6*	*X7*	*X8*	*X9*
LOG-LIN	0.01	0.00	0.01	0.00	−0.10	0.00	0.00	0.10	0.30
**Elasticities of the CONSUMPTION/PRICE Model (Primary Sector)**									
	*X1*	*X2*	*X3*	*X4*	*X5*	*X6*	*X7*	*X8*	*X9*
LOG-LIN	0.00	3.41	−3.01	1.21	−2.11	2.81	1.50	8.52	−4.51

**Table 14:** Equations of functional forms of econometric models PRICE/PRICE (MEAT—secondary sector).

Type of the Formulation	Equations
LIN	*Yc* = 62.017 − 0.062 * *X1c* − 0.001 * *X2c* + 0.439 * *X3* + 0.181 * *X4* + 0.002 * *X5*
LOG-LIN	LNYc = 4.235 − 0.001 * *X1c* − 0.000008301 * *X2c* + 0.004 * *X3* + 0.002 * *X4* + 0.00001291 * *X5*
LIN-LOG	*Yc* = − 69.344 – 6.371 * ln*X1c* − 0.951 * ln*X2c* + 43,756 * ln*X3* + 1.485 * ln*X4* + 0.249 * ln*X5*
LOG	LNYc = 2.956 − 0.063 * ln*X1c* − 0.01 * ln*X2c* + 0.428 * ln*X3* + 0.015 * ln*X4* + 0.002 * ln*X5*

**Table 15:** Comparison of the calculated elasticities or determination of the opposite elasticities in the econometric models PRICE/PRICE and CONSUMPTION/PRICE (meat—secondary sector).

Elasticities in the PRICE/PRICE Model (Secondary Sector)					
	*X1*	*X2*	*X3*	*X4*	*X5*
LIN	−0.06	−0.01	0.44	0.01	0.00
**Elasticities of the CONSUMPTION/PRICE Model (Secondary Sector)**					
	*X1*	*X2*	*X3*	*X4*	*X5*
LOG	6.89	2.00	−1.57	−1.56	0.59

**Table 16:** Equations of functional forms of econometric models PRICE/PRICE (MEAT—tertiary sector).

Type of the Formulation	Equations
LIN	*Yc* = 38.508 − 0.001 * *X1c* + 0.001 * *X2c* − 0.01 * *X3c* + 0.009 * *X4c* + 0.568 * *X5* − 0.037 * *X6* + 0.093 * *X7*
LOG-LIN	LNYc = 4.000 − 0.00001188 * *X1c* + 0.000008777 * *X2c* − 0.00009817 * *X3c* + 0.00008861 * *X4c* + 0.006 * *X5* + 0.0 * *X6* + 0.001 * *X7*
LIN-LOG	*Yc* = − 184.493 − 1.432 * ln*X1c* + 0.806 * ln*X2c* − 1.072 * ln*X3c* + 0.884 * ln*X4c* + 57,242 * ln*X5* – 3.566 * ln*X6* + 9.348 * ln*X7*
LOG	LNYc = 1.804 − 0.014 * ln*X1c* + 0.008 * ln*X2c* − 0.011 * ln*X3c* + 0.008 * ln*X4c* + 0.566 * ln*X5* − 0.036 * ln*X6* + 0.09 * ln*X7*

**Table 17:** Comparison of the calculated elasticities and determination of the opposite elasticities in the econometric models PRICE/PRICE and CONSUMPTION/PRICE (meat—tertiary sector).

Elasticities in the PRICE/PRICE Model (Tertiary Sector)							
	*X1*	*X2*	*X3*	*X4*	*X5*	*X6*	*X7*
LIN	−0.01	0.01	−0.01	0.01	0.57	−0.04	0.09
**Elasticities of the CONSUMPTION/PRICE Model (Tertiary Sector)**							
	*X1*	*X2*	*X3*	*X4*	*X5*	*X6*	*X7*
LOG	0.16	1.10	−2.74	−0.65	6.11	−5.67	−13.22

**Table 18:** Level of maximum sensitivity to price changes in agricultural and food products for changes in cost levels within a single sector in the MESO model.

Maximum Sensitivity for Price Changes in Agricultural Products in the Event of Cost Changes in a Single Sector (Calculated Weights—in %)		
Primary Sector	Secondary Sector	Tertiary Sector
32.7	55.2	12.1

**Table 19:** Equations of functional forms of econometric models PRICE/PRICE (MILK, CHEESE AND EGGS—primary sector).

Type of the Formulation	Equations
LIN	*Yd* = 54.589 + 0.001 * *X1d* + 0.095 * *X2* + 0.002 * *X3* + 0.007 * *X4* + 0.113 * *X5* + 0.016 * *X6* + 0.086 * *X7* + 0.097 * *X8* + 0.027 * *X9*
LOG-LIN	LNYd = 4.166 + 0.000009831 * *X1d* + 0.001 * *X2* + 0.00001766 * *X3* + 0.00006249 * *X4* + 0.001 * *X5* + 0.0 * *X6* + 0.001 * *X7* + 0.001 * *X8* + 0.0 * *X9*
LIN-LOG	*Yd* = − 122.215 +1.241 * ln*X1d* + 9,489 * ln*X2* + 0.11 * ln*X3* + 0.986 * ln*X4* + 11.188 * ln*X5* + 1.687 * ln*X6* + 9,587 * ln*X7* + 10.474 * ln*X8* + 2.842 * ln*X9*
LOG	LNYd = 2.456 + 0.012 * ln*X1d* + 0.093 * ln*X2* + 0.001 * ln*X3* + 0.009 * ln*X4* + 0.105 * ln*X5* + 0.017 * ln*X6* + 0.094 * ln*X7* + 0.097 * ln*X8* + 0.031 * ln*X9*

**Table 20:** Comparison of calculated elasticities or determination of opposite elasticities in the econometric models PRICE/PRICE and CONSUMPTION/PRICE (MILK, CHEESE, EGGS—primary sector).

Elasticities in the PRICE/PRICE Model (Primary Sector)									
	*X1*	*X2*	*X3*	*X4*	*X5*	*X6*	*X7*	*X8*	*X9*
**LOG-LIN**	0.01	0.10	0.00	0.01	0.10	0.00	0.10	0.10	0.00
**Elasticities of the CONSUMPTION/PRICE Model (Primary Sector)**									
	*X1*	*X2*	*X3*	*X4*	*X5*	*X6*	*X7*	*X8*	*X9*
**LIN-LOG**	−60.70	313.53	177.59	65.43	−38.54	−61.22	−101.77	−1105.87	−2340.50

**Table 21:** Equations of the functional forms of econometric models PRICE/PRICE (MILK, CHEESE AND EGGS—secondary sector).

Type of the Formulation	Equations
LIN	*Yd* = 94.7 + 0.023 * *X1d* + 0.0 * *X2d* + 0.002 * *X3* + 0.306 * *X4* + 0.01 * *X5*
LOG-LIN	LNYd = 4.552 + 0.0 * *X1d* − 0.000002038 * *X2d* + 0.00003454 * *X3* + 0.003 * *X4* + 0.0 * *X5*
LIN-LOG	*Yd* = 66.901 + 3.349 * ln*X1d* − 0.053 * ln*X2d* + 1.702 * ln*X3* + 2.231 * ln*X4* + 1.303 * ln*X5*
LOG	LNYd = 4.273 + 0.033 * ln*X1d* − 0.001 * ln*X2d* + 0.018 * ln*X3* + 0.022 * ln*X4* + 0.013 * ln*X5*

**Table 22:** Comparison of the calculated elasticities and determination of the opposite elasticities in the econometric models PRICE/PRICE and CONSUMPTION/PRICE (MILK, CHEESE, EGGS—secondary sector).

Elasticities in the PRICE/PRICE Model (Secondary Sector)					
	*X1*	*X2*	*X3*	*X4*	*X5*
**LIN**	0.02	0.00	0.00	0.02	0.01
**Elasticities of the CONSUMPTION/PRICE model (secondary sector)**					
	*X1*	*X2*	*X3*	*X4*	*X5*
**LIN-LOG**	2182.746	−320.970	4182.105	−360.963	−334.491

**Table 23:** Equations of functional forms of econometric models PRICE/PRICE (MILK, CHEESE AND EGGS—tertiary sector).

Type of the Formulation	Equations
LIN	*Yd* = 8.966 + 0.001 * *X1d* − 0.001 * *X2a* − 0.005 * *X3d* − 0.01 * *X4d* + 0.976 * *X5* + 0.061 * *X6* − 0.108 * *X7*
LOG-LIN	LNYd = 3.72 + 0.00000777 * *X1d* − 0.00001275 * *X2a* − 0.00003787 * *X3d* + 0.0 * *X4d* + 0.009 * *X5* + 0.001 * *X6* − 0.001 * *X7*
LIN-LOG	*Yd* = − 322.039 + 1.143 * ln*X1d* − 1.645 * ln*X2a* − 0.617 * ln*X3d* − 0.946 * ln*X4d* + 97,436 * ln*X5* + 6.308 * ln*X6* – 9.828 * ln*X7*
LOG	LNYd = 0.5 + 0.011 * ln*X1d* − 0.016 * ln*X2a* − 0.005 * ln*X3d* − 0.01 * ln*X4d* + 0.943 * ln*X5* + 0.06 * ln*X6* − 0.09 * ln*X7*

**Table 24:** Comparison of the calculated elasticities or determination of the opposite elasticities in the econometric models PRICE/PRICE and CONSUMPTION/PRICE (MILK, CHEESE, EGGS—tertiary sector).

Elasticities in the PRICE/PRICE Model (Tertiary Sector)							
	*X1*	*X2*	*X3*	*X4*	*X5*	*X6*	*X7*
**LOG-LIN**	0.01	−0.01	0.00	0.00	0.90	0.10	−0.10
**Elasticities of the CONSUMPTION/PRICE Model (Tertiary Sector)**							
	*X1*	*X2*	*X3*	*X4*	*X5*	*X6*	*X7*
**LOG**	1.56	−0.53	−0.71	1.72	−3.59	−6.65	−4.777

**Table 25:** Level of maximum sensitivity to price changes in agricultural products for changes in cost levels within a single sector in the MILK, CHEESE, EGGS model.

Maximum Sensitivity for Price Changes in Agricultural Products in the Event of Cost Changes in a Single Sector (Calculated Weights—in %)		
Primary Sector	Secondary Sector	Tertiary Sector
23.3	2.6	74.1

## Data Availability

The original contributions presented in the study are included in the article, further inquiries can be directed to the corresponding author.
